# Deconstructing the Polymerase Chain Reaction: Understanding and Correcting Bias Associated with Primer Degeneracies and Primer-Template Mismatches

**DOI:** 10.1371/journal.pone.0128122

**Published:** 2015-05-21

**Authors:** Stefan J. Green, Raghavee Venkatramanan, Ankur Naqib

**Affiliations:** 1 DNA Services Facility, Research Resources Center, University of Illinois at Chicago, Chicago, Illinois, United States of America; 2 Dept. of Biological Sciences, University of Illinois at Chicago, Chicago, Illinois, United States of America; 3 Dept. of Bioengineering, University of Illinois at Chicago, Chicago, Illinois, United States of America; Texas A&M University, UNITED STATES

## Abstract

The polymerase chain reaction (PCR) is sensitive to mismatches between primer and template, and mismatches can lead to inefficient amplification of targeted regions of DNA template. In PCRs in which a degenerate primer pool is employed, each primer can behave differently. Therefore, inefficiencies due to different primer melting temperatures within a degenerate primer pool, in addition to mismatches between primer binding sites and primers, can lead to a distortion of the true relative abundance of targets in the original DNA pool. A theoretical analysis indicated that a combination of primer-template and primer-amplicon interactions during PCR cycles 3–12 is potentially responsible for this distortion. To test this hypothesis, we developed a novel amplification strategy, entitled “Polymerase-exonuclease (PEX) PCR”, in which primer-template interactions and primer-amplicon interactions are separated. The PEX PCR method substantially and significantly improved the evenness of recovery of sequences from a mock community of known composition, and allowed for amplification of templates with introduced mismatches near the 3’ end of the primer annealing sites. When the PEX PCR method was applied to genomic DNA extracted from complex environmental samples, a significant shift in the observed microbial community was detected. Furthermore, the PEX PCR method provides a mechanism to identify which primers in a primer pool are annealing to target gDNA. Primer utilization patterns revealed that at high annealing temperatures in the PEX PCR method, perfect match annealing predominates, while at lower annealing temperatures, primers with up to four mismatches with templates can contribute substantially to amplification. The PEX PCR method is simple to perform, is limited to PCR mixes and a single exonuclease step which can be performed without reaction cleanup, and is recommended for reactions in which degenerate primer pools are used or when mismatches between primers and template are possible.

## Introduction

To target single gene fragments from multiple organisms within a complex community of known and unknown organisms using PCR has required careful bioinformatics analyses and empirical testing of many primers. Ideal criteria for primers include: (i) primers should match genes of all known organisms within the group of interest, and should be able to target genes from unknown organisms in sub-taxonomic levels (*i*.*e*. domain-level primers targeting Bacteria should be conserved among all known bacteria, with the assumption that unknown bacteria will also contain these conserved regions), (ii) primer pairs should be balanced for melting temperature and produce robust amplification, and (iii) primers need to span one or more hyper-variable regions of the gene. Even in highly conserved regions of the ribosomal RNA (rRNA) genes, no primer set matches these criteria perfectly, although many excellent primer sets have been developed (e.g., [1,2–5]). To increase target range, pools of primers (degenerate primers) are used. Ribosomal RNA genes are the preferred targets for broad-spectrum analyses as the level of genetic diversity in conserved regions of rRNA genes is lower than that present in functional genes where amino acid sequences can be highly conserved even in the presence of substantial DNA-level changes due to the degeneracy of the genetic code. Therefore, degenerate primers used for PCR amplification of rRNA genes generally have lower levels of degeneracy than primers used for amplification of functional genes.

Amplicon sequencing approaches for microbial surveys, including those targeting rRNA genes, are limited in several ways: (i) amplicon sequencing studies of mixed microbial communities fundamentally distort the “true” structure of the community through systematic bias associated with input genomic DNA (gDNA) concentration, differential amplification efficiency due to mismatches between primer and template, differential amplification efficiency within mixtures of degenerate primer pools and varying melting temperature, through differential consumption of specific primers in degenerate primer pools during later cycles of PCR, through PCR saturation during late PCR stages, and through preferential amplification of specific targets [6–14]; (ii) even degenerate primer pools are not degenerate enough to target all the intended targets [13], and this is particularly true for many functional genes; (iii) ribosomal RNA genes come with a built-in bias: namely, the copy number variation from organism to organism distorts the observed structure of the community, and favors organisms with high copy number (this has been partially addressed in [15]); and (iv) quantitative analyses are even more sensitive to varying amplification efficiencies of degenerate primers as high efficiency of PCR amplification is necessary, and distortions may occur if the quantitative calibration standards have no mismatches between primer and target, while environmental samples contain mixtures of perfectly matching and single- and multiple-mismatch targets. Taken together, direct PCR-sequencing and quantitative PCR analysis of microbial rRNA (and other) genes is certainly providing a distorted composition and structure, and some taxa are not detected at all [16].

This study describes a new methodological approach to PCR amplification to address distortions due to variable primer melting temperature in degenerate primer pools and distortions arising from mismatches between the primer and template sequence. The method also provides a mechanism to determine which primers from a primer pool are involved in annealing to gDNA templates, and what number of mismatches can be tolerated by a given primer pair.

To validate the technique, a standard PCR assay targeting the V4 variable region of microbial SSU rRNA genes was employed. This assay has been previously described [2,17], and is employed by the Earth Microbiome Project (EMP). The primer set (515F and 806R), targeting Bacteria and Archaea, has found wide acceptance in part due to an appropriate amplicon length for sequencing on the Roche 454, Illumina MiSeq and Ion Torrent Personal Genome Machine (PGM) next-generation sequencing (NGS) platforms. The primer set is highly degenerate (2-fold degeneracy in the 515F primer pool, 18-fold degeneracy in the 806R primer pool). The new methodology was optimized using DNA from a constructed mock community. After optimization, complex environmental gDNA samples were analyzed using the new method and the results compared with those generated with standard PCR methods. The method, termed polymerase-exonuclease (PEX) PCR, significantly improved the evenness of recovery of templates in a mock simplified community, and had a significant effect on the observed structure of complex microbial communities.

## Materials and Methods

### DNA templates

Genomic DNA was extracted from fecal pellets of domesticated chinchillas (“Chin”) using the Tissue DNA Purification Kit and Maxwell 16 System (Promega Corporation, Madison, WI). No specific permissions were required for the collection of the chinchilla feces. Genomic DNA was extracted from multiple sediment samples from Lake Huron (Lat/Long coordinates: 44°05.9933 N, 082°30.1474; 44°19.9650 N, 082°49.9548 W) using the PowerSoil DNA extraction kit (Mo Bio Laboratories, Carlsbad, CA), and pooled to make a complex gDNA pool (“Sed”). All lake sediment samples were collected on the RV Lake Guardian operated by the EPA, and no sampling permits were required. The field studies did not involve endangered or protected species. DNA quantity was measured using a Qubit 2.0 fluorometer with the dsDNA BR Assay (Life Technologies, Grand Island, NY). Artificial double-stranded DNAs (gBlocks Gene Fragments) were synthesized by Integrated DNA Technologies, Inc. (IDT; Coralville, Iowa). The synthesized 492 bp gene fragments were derived from a portion of the SSU rRNA gene of *Rhodanobacter denitrificans* 2APBS1 [18,19], covering the 515F and 806R primer positions with approximately 75 bp on either side of the targeted region. The sequence of the primer annealing sites was altered to introduce mismatches with primers or to match only a single primer from the degenerate primer pool. To allow for identification of each of the four gene fragments (*i*.*e*., Mock A, B, C and D) after sequencing, a 10-bp region in the center of each of the fragments was scrambled to create a unique identifier sequence (S1 File). Other than alterations in the primer sites and the 10-bp region in the middle of the gene fragment, the sequences were identical. The exact primer site sequences and number of mismatches with each of the primers in the degenerate pool are shown in S2 File. The gene fragments were delivered as 200 ng stocks, and were dissolved in 20 microliters of TE buffer, yielding solutions with 10 ng/ul (~1.85 x 10^10^ copies/ul). A single equimolar pool of the four synthesized double-stranded gene fragments was made (“Mock”), and a 1/100^th^ dilution of this pool was used for all subsequent analyses.

### Standard PCR (Targeted amplicon sequencing, TAS)

**Fig 1:**
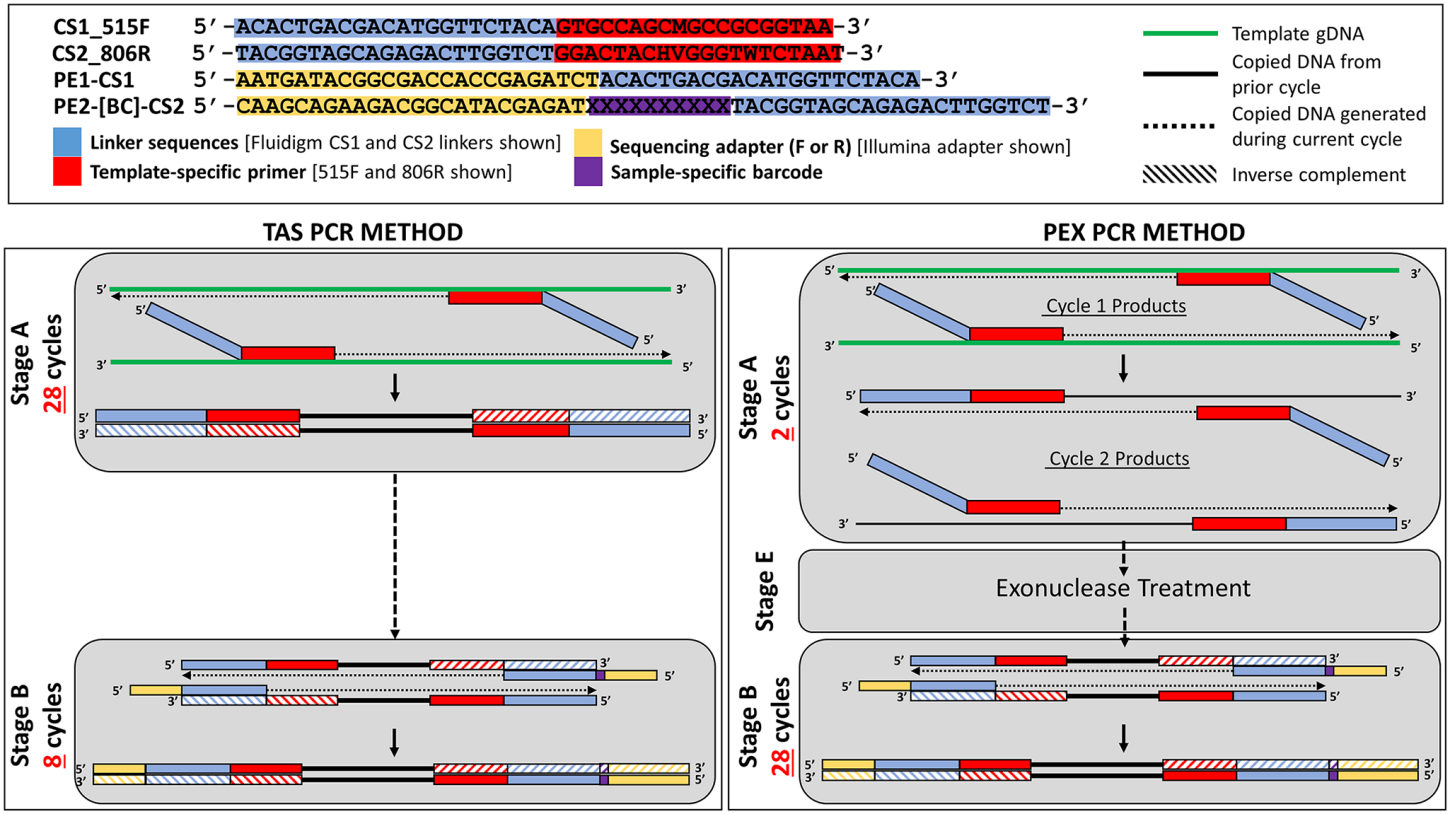
Schematic of Targeted amplicon sequencing (TAS) and Polymerase Exonuclease (PEX) PCR methods.

The benchmark or standard PCR was a targeted-amplicon sequencing (TAS) approach, similar to that described by Bybee et al. [20] and de Carcer et al. [21]. Briefly, genomic DNA is amplified using primers targeting the gene of interest, but containing 5’ linker sequences which do not anneal to the genomic DNA template. Subsequently, the amplicons generated during the first stage of PCR are use as template for amplification with primers containing sequencing adapters, sample-specific barcode sequences, and the same linker sequences, but located at the 3’ end of the primer (TAS PCR method, Fig 1; S1 Fig). In this study, TAS sequencing was performed in two stages (“A” and “B”) of 28 cycles and 8 cycles, respectively, generating amplicons ready for sequencing on an Ion Torrent Personal Genome Machine (PGM) sequencer or Illumina MiSeq. Unless specified, gDNA was PCR amplified with primers CS1_515F and CS2_806R (Primer set 2; Table 1). The primers contained 5’ sequence tags (known as common sequence 1 and 2, CS1 and CS2) as described previously [22]. PCR amplifications were performed in 10 microliter reactions. A master mix for each reaction was made using the 2X AccuPrime SuperMix II (Life Technologies). The final concentration of CS1_515F and CS2_806R primers was 500 nM. Approximately 25 ng of environmental gDNA or 1 μl of the “mock” DNA was added to each reaction. Cycling conditions were as follows: 95°C for 5 minutes, followed by 28 cycles of 95°C for 30”, variable annealing temperature for 45” and 68°C for 30”. A final, 7 minute elongation step was performed at 68°C.

**Table 1:** Primers used in this study.

Primer Set	Primer Name	Sequences (5'-3')*	Reference
1	515F	GTGCCAGC**M**GCCGCGGTAA	Caporaso et al. 2010
	806R	GGACTAC**HV**GGGT**W**TCTAAT	Caporaso et al. 2010
2	CS1_515F	ACACTGACGACATGGTTCTACA-GTGCCAGC**M**GCCGCGGTAA	Caporaso et al. 2010; This study
	CS2_806R	TACGGTAGCAGAGACTTGGTCT-GGACTAC**HV**GGGT**W**TCTAAT	Caporaso et al. 2010; This study
3	CS1_515F	ACACTGACGACATGGTTCTACA-GTGCCAGC**M**GCCGCGGTAA	Caporaso et al. 2010; This study
	CS2_806R-NI	TACGGTAGCAGAGACTTGGTCT-GGACTAC**55**GGGT**W**TCTAAT	Caporaso et al. 2010; This study
4	Ion Torrent A Adapter—Barcode—CS2	CCATCTCATCCCTGCGTGTCTCCGACTCAG-**[BC]**-TACGGTAGCAGAGACTTGGTCT	Fluidigm
	Ion Torrent P1 Adapter—CS1	CCTCTCTATGGGCAGTCGGTGAT-ACACTGACGACATGGTTCTACA	Fluidigm
5	Illumina PE1—CS1	AATGATACGGCGACCACCGAGATCT-ACACTGACGACATGGTTCTACA	Fluidigm
	Illumina PE2—Barcode—CS2	CAAGCAGAAGACGGCATACGAGAT-**[BC]**-TACGGTAGCAGAGACTTGGTCT	Fluidigm

* Underlined sequences represent the common sequence linkers. M = A or C; H = A or C or T; V = A or C or G; W = A or T; 5 = 5-nitroindole substitution. **[BC]** = 10 base barcode that is unique to each sample.

A second PCR amplification (Stage B; Fig 1; S1 File), used to incorporate barcodes and sequencing adapters into the final PCR product, was performed in 10 microliter reactions, using the same master mix conditions as described above. Each well received a separate primer pair containing a unique 10-base barcode, obtained from the Fluidigm Access Array Barcode Library for Ion Torrent or Illumina Sequencers (Primer set 4 or 5; Table 1). The final concentration of each primer was 400 nM. Cycling conditions were as follows: 95°C for 5 minutes, followed by 8 cycles of 95°C for 30”, 60°C for 30” and 68°C for 30”. A final, 7 minute elongation step was performed at 68°C.

### Specialty amplification reactions (PEX PCR method)

An amplification strategy was developed to reduce amplification bias. This novel strategy, entitled PEX PCR (Polymerase-EXonuclease PCR), also included the two polymerase-mediated stages with the same primer sets as described above (PEX PCR method, Fig 1; S1 File **)**. Primer concentration during the first stage was reduced to 125 nM. The first stage (stage “A”) consisted of only 2 cycles, and the second amplification reaction (Stage “B”; Fig 1) consisted of 28 cycles. Otherwise, reactions were set up identically as described above. Tubes containing master mix, gDNA or “mock” DNA, and CS1_515F and CS2_806R primers were heated to 95°C for five minutes and then to the specified annealing temperature (30°C, 35°C, 40°C, 45°C, 50°C, or 55°C) for 20 minutes. This cycle was repeated once more (two cycles total). In control reactions used to determine if the stage “A” primers were active during the stage “B” reactions (Fig 1), only a single stage “A” cycle was performed.

After stage “A”, samples were either directly treated with exonuclease I or diluted 1/10^th^ in sterile water (Mo Bio Laboratories), and treated with exonuclease I (Stage “E”). Some trial reactions did not receive exonuclease treatment. Exonuclease digestion was performed with five microliters of diluted or undiluted sample from the first two-cycle stage of the PEX method. Five μl of sample were mixed with two μl of ExoSAP (ExoSAP-IT For PCR Product, Affymetrix, Santa Clara, CA) and incubated according to the manufacturer’s instructions (37°C for 15’, 80°C for 15’). Three microliters of the ExoSAP-treated sample was transferred to the second PCR reaction containing primer sets 4 or 5 (Table 1), as described above. Each sample and replicate received a primer set with a unique barcode. PCR was performed as described above, but with 28 cycles instead of 8. In tests without exonuclease treatment, 1 μl of sample from the first two-cycle stage of PEX PCR method was transferred to the second 28-cycle stage of the reaction.

In addition, some reactions were performed with a modified 806R primer (“806R-NI”; Primer set 3, Table 1). The most highly degenerate positions of the 806R primer (two adjacent positions are 3-fold degenerate, “H” and “V”, Table 1) were replaced with so-called “universal base” 5-nitroindole substitutions [23]. All primers were synthesized by IDT. PCR conditions were not altered when 806R-NI primers were utilized.

### Next-generation amplicon sequencing

Final PCR amplicons from TAS and PEX PCR methods were pooled in equal volume and purified using solid phase reversible immobilization (SPRI) cleanup, using AMPure XP beads (Beckman Coulter, Brea CA) at a ratio of 0.6X (v:v) SPRI solution to sample. Final quality control was performed using the D1000 ScreenTape assay implemented on a TapeStation2200 instrument (Agilent Technologies, Santa Clara, CA) and Qubit analysis. The pooled libraries were quantitated by qPCR using a library quantification kit (KAPA Biosystems, Wilmington, MA). For Illumina sequencing, the library pool was spiked with 15% non-indexed PhiX control library provided by Illumina and then loaded onto a MiSeq v2 flow cell at a concentration of 8 pM for cluster formation and sequencing (paired-end reads, 2x250 bases). Custom sequencing and index read primers (according to Fluidigm Access Array Illumina sequencing guide) were added to the appropriate wells of the reagent cartridge. Illumina MiSeq sequencing was performed at the W.M. Keck Center for Comparative and Functional Genomics at the University of Illinois at Urbana-Champaign, and data were analyzed using the Casava1.8 pipeline. For Ion Torrent sequencing, pooled libraries were diluted to 8.5 pM for emulsion PCR. Libraries were prepared for sequencing using automated emulsion PCR employing the Ion PGM template OT2 400 kit. Sequencing was performed on an Ion Torrent Personal Genome Machine (PGM) with a 318 chip, using the Ion PGM sequencing 400 kit (Life Technologies). Barcode sequences from Fluidigm were provided to the PGM server, and sequences were automatically binned according to 10-base multiplex identifier (MID) sequences. Raw reads were recovered from the PGM server as FASTQ files. Ion Torrent PGM sequencing was performed at the DNA Services Facility at the University of Illinois at Chicago.

### Analysis of mock community amplicon sequence data

Under ideal conditions, PCR amplification of the mock community would result in equal amplification of the four templates, resulting in 25% of all sequence reads attributed to each of the four target templates. To assess the effectiveness of each modification to the amplification reactions, FASTQ files (Ion Torrent and Illumina) were imported into the software package CLC genomics workbench 7.0 (CLC Bio, Qiagen, Boston, MA). Raw sequence data were trimmed using quality trimming algorithms (quality threshold, 0.05 for Ion Torrent and 0.01 for Illumina), and common sequences (Ion Torrent only). Sequence data from the “mock” communities were mapped against the four variants of *R*. *denitrificans* 2APBS1 gene (S1 File) within the software package CLC Genomics workbench. Counts for each variant were generated.

Divergence from equal relative abundance was calculated by summing the difference between expected abundance (*e*.*g*., 25% for each variant) and measured abundance for each variant (“Ideal score (IS)”; $\sum_{i=1}^{n}abs(1/n-Pi)*100$ where n = number of targets in equimolar pool and P*i* = percentage of NGS sequencing reads mapping to target i). Ideal scores were calculated for all four possible templates (IS4), for three target templates (excluding the two-mismatch template; IS3), and for the two no-mismatch templates (IS2). Lower scores represent a closer representation of the ideal (for evenly distributed templates, potential scores range from 0 to 2*(100/n)*(n-1)).

### Analysis of environmental community amplicon sequence data

For analysis of amplicons generated from environmental gDNA, sequences were initially processed in CLC genomics workbench. Illumina reads were merged, and subsequently quality-trimmed to remove low-quality reads. Ion Torrent data were quality trimmed only, and reads of less than 200 bases were removed from the analysis. For analysis of microbial community structure, primer sequences (515F and 806R) were removed. The remaining sequences were exported as FASTA files and processed with the software package QIIME [24]. Briefly, sequences were screened for chimeras using the usearch61 algorithm [25], and putative chimeric sequences were removed from the dataset. Subsequently, each sample sequence set was sub-sampled to the smallest sample size to avoid analytical issues associated with variable library size [26]. Sub-sampled data were pooled and renamed, and clustered into operational taxonomic units (OTU) at 97% similarity. Representative sequences from each OTU were extracted, and these sequences were classified using the “assign_taxonomy” algorithm implementing the RDP classifier, with the Greengenes reference OTU build [27,28]. A biological observation matrix (BIOM; [29]) was generated at taxonomic levels from phylum to genus using the “make_OTU_table” algorithm. The BIOMs were imported into the software package Primer6 [30] for analysis and visualization. Figures were generated using the software package OriginPro8.5 (OriginLab Corporation, Northampton, MA) and in the software packages Excel and Powerpoint (Microsoft, Redmond, Washington).

### Analysis of primer utilization patterns

In standard bioinformatics analyses, sequence data at primer sites are removed prior to bioinformatics analyses. To examine which primers annealed to template strands in TAS and PEX PCR reactions, quality-trimmed sequences containing primer sequences were exported from CLC genomics as FASTA files, or mapped to mock community reference sequences, and subsequently exported. Sequences were imported into Excel, and searching algorithms were implemented to detect each primer variant from the degenerate primer pools. For reactions in which the 806R primer contained 5-nitroindole substitutions (*i*.*e*. primer set 3, Table 1), an additional 14 potential variants for the 806R primer were examined, since any base can potentially be incorporated opposite to a 5-nitroindole substitution.

### Data Access

The gene amplicon sequence data generated as part of this study have been submitted to the NCBI BioProject database under accession number PRJNA262579. Sample details and FASTQ file names are provided in S3 File.

## Results

### Theory

**Fig 2:**
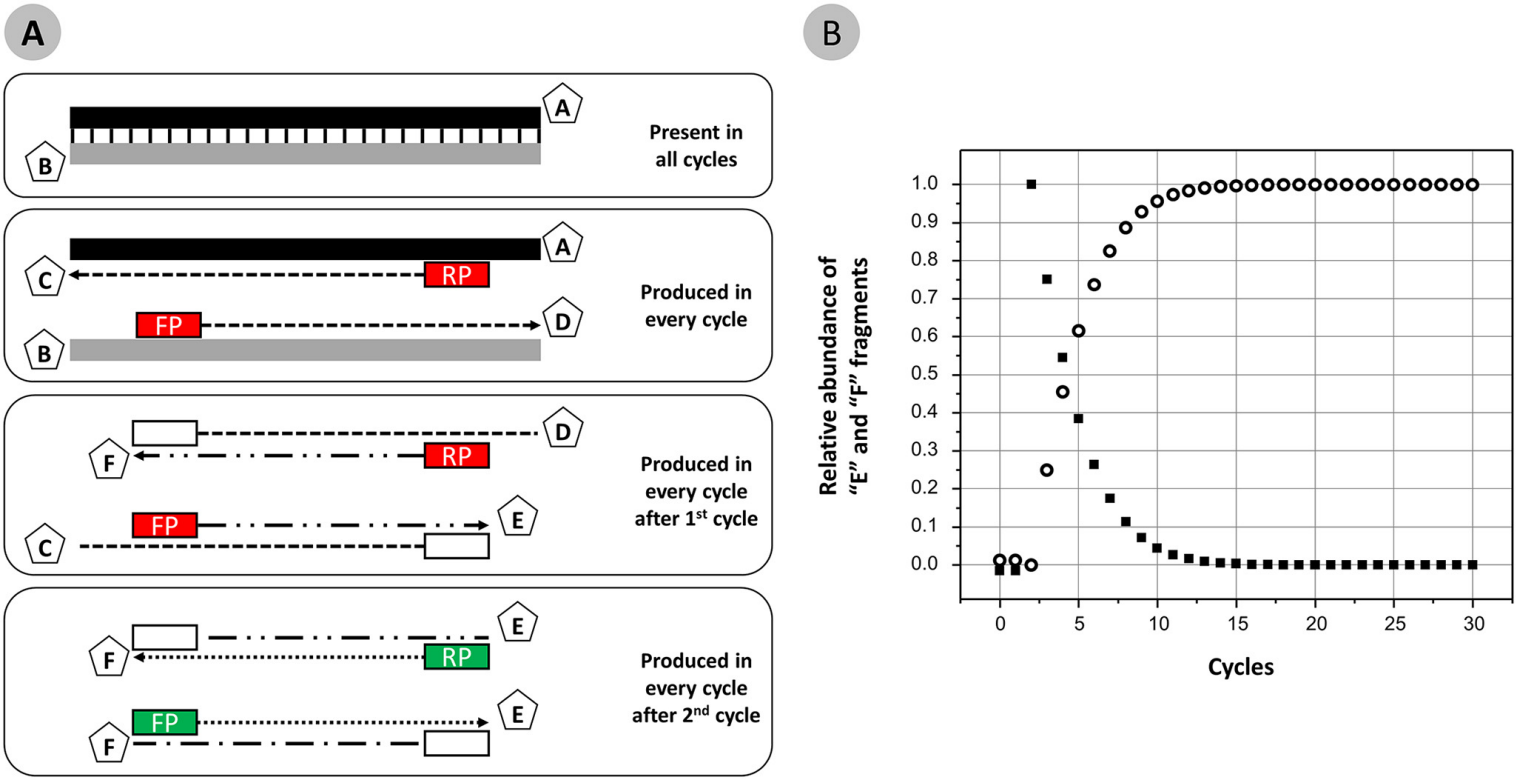
Types and abundance of DNA fragments found in PCR. **(A)** Template DNA fragments (containing strands “A” and “B”) are added to PCR reactions and are conserved throughout the reaction. Fragments “A” and “B” serve as templates for copying in each cycle, with hybrid molecules “C” and “D” produced in a linear fashion each cycle. In cycles two and above, “C” and “D” are copied, creating hybrid molecules “E” and “F” in a linear fashion. In cycles three and above, the “E” and “F” fragments generated in prior cycles are copied into inverse complement fragments “F” and “E”, respectively, in an exponential fashion. Red boxes indicate ‘natural’ primer annealing to genomic DNA template or copy of gDNA template. Green boxes indicate ‘artificial’ primer annealing to primer sites that are copies of oligonucleotide primers added to the PCR mixture, and incorporated during previous cycles. **(B)** The relative abundance of “E” and “F” fragments generated by ‘natural’ template-primer interactions (“C”,”D” → “E”,”F”; shown as solid squares) and by artificial template-primer interactions (“E”,”F” → “F”,”E”; shown as open circles) varies by cycle. At the end of cycle two, all “E” and “F” fragments have been generated only by ‘natural’ template-primer interactions.

During the polymerase chain reaction, two distinct types of primer-template interactions can occur, and these types of interactions likely operate at different efficiencies. The first primer-template interaction is that of the PCR primers with the source genomic DNA; such interactions include both perfect matches and primer annealing to regions containing mismatches of unpredictable number and location. Subsequently, however, PCR primers interact with primer sites that have been created during the PCR cycle, and are the inverse complement of the synthesized oligonucleotides used as PCR primers. Here, the primer-template interactions can also include perfect matches and mismatches, but the scale of mismatch annealing is proportional to the number of degeneracies in the primer pool. We have termed primer annealing to genomic DNA as “natural” interactions and primer annealing to PCR amplicons as “artificial” interactions (Fig 2A).

A critical observation of these two type of primer-template interactions is that there are two mechanisms for generating the final fragments generated by PCR (*i*.*e*., “E” and “F” fragments). This includes natural template-primer annealing processes (*i*.*e*. “E” and “F” fragments from “C” and “D” fragments) and artificial template-primer annealing processes (*i*.*e*. “E” and “F” fragments from other “F” and “E” fragments) (Fig 2A). Artificial template-primer interactions occur only in cycles three and above, since no “E” and “F” molecules exist in the reaction until the end of cycle two. Regardless of the degeneracy of the primer pool, natural template-primer interactions are likely to be more complex due to the great potential for the presence of primer-template mismatches as a result of the underlying genetic diversity at primer sites. Artificial template-primer interactions are less complex, and the number of potential mismatches between primer and template is proportional to the diversity of the primer pool employed.

Artificial template-primer interactions ultimately dominate PCR as artificial template-primer interactions yield exponential amplification, while natural template-primer annealing interactions yield linear amplification. During the early cycles of PCR (*i*.*e*., up to the 12^th^ cycle), however, natural template-primer annealing interactions can contribute substantially (>1%) to the overall pool of fragments that are the final yield of PCR (Fig 2B), assuming both processes operate at 100% efficiency.

At the end of cycle 2, however, no exponential amplification can have occurred. In fact, there has (providing that the reaction operates at 100% efficiency) been a non-destructive conversion of gene targets present in large gDNA fragments to short gene fragments bounded at either end by the forward and reverse primer sites. These fragments are not true double-strands, but hybrids of one long strand with only one primer site, and a short strand with both primer sites present (Third box, Fig 2A). In theory, for every gDNA copy of double-stranded DNA there exist two single stranded fragments of the same region. Thus, the end of the second cycle of PCR marks a critical transition from linear copying only (cycles 1 and 2) to linear and exponential amplification (cycles 3 and above).

In this study, ‘natural’ template-primer interactions were separated from ‘artificial’ template-primer interactions. To achieve this separation, PCR primers with 5’ linker sequences which do not interact with the native genomic template DNA were synthesized (*e*.*g*., Primer Set 2, Table 1; Fig 1). These linkers are intended to have no similarity to any known biological sequences. Thus, at the end of cycle two, E and F fragments are bounded by the two separate linkers (a “forward” linker and a “reverse” linker). After the second cycle is complete, stage “A”of the overall reaction is terminated, and the original primers are removed using DNA exonuclease I digestion. The gDNA (A,B fragments), first and second cycle copies (C,D fragments) and second-cycle only copies (E,F fragments) are then transferred to a second PCR in which new primers, targeting the linker sequences, are used instead of template-specific sequences (Fig 1, S1 Fig). Only the “E” and “F” fragments are suitable templates for such amplification. The purpose of this is to perform the minimum number of cycles with degenerate primers operating under ‘natural’ template-primer interaction conditions and rapidly shift the exponential amplification of amplicons to ‘artificial’ template-primer interactions employing non-degenerate primers. Thus, the template-specific primers are not involved in amplification. In this way, bias associated with degenerate primers and with template-primer mismatches may be reduced.

### Application of a new pipeline for PCR amplification of templates

To separate ‘natural’ and ‘artificial’ template-primer interactions, a simple workflow, customizable to any primer set, was developed (Fig 1, S1 Fig). After two cycles of denaturation, annealing and extension, the reaction was terminated (Stage “A”). Subsequently, the sample incubated with an Exonuclease I to digest single stranded DNA (*i*.*e*., unincorporated primers). The lowered initial primer concentrations (125 nM) were used since the exonuclease activity was insufficient to remove primers from reactions with higher concentration. In some cases, stage 1 cycling products were diluted 1/10^th^ prior to exonuclease digestion. Exonuclease activity in the Exo-SAP product is optimized for post-PCR conditions in which much of the primer pool is consumed in the amplification of the template. Since no amplification occurs in the first two cycles of the PEX PCR method, only a small amount of primer is consumed in the reaction. We initially observed this by testing for second stage amplification after only one cycle instead of two. We anticipated that after one cycle only, the second stage amplification could not occur as no template molecules would contain both the forward and reverse common sequences (*i*.*e*., no E and F fragments would be produced before cycle 2). When amplification was observed after only 1 cycle, this was taken to indicate that left-over primer from the first stage of the reaction was still active during the second stage of the reaction, generating additional copies during second stage cycling. This was subsequently verified when examining primer utilization patterns (see below). Thus, conditions were optimized until stage “B” PCR produced no amplicons when only one cycle of stage “A” reaction was performed. For the primer set CS1_515F and CS2_806R, these conditions could be met by lowering the initial primer concentration to 125 nM (since most of the primer is not used in the first two cycles), and subsequently diluting the reaction 1/10^th^ before Exo-SAP treatment. Although manufacturer’s details (*i*.*e*. Exo-SAP can degrade 5 μM of primer) suggest that this dilution is not necessary, we often observed stage “B” (Fig 1) amplification without dilution. Finally, exonuclease I is a 3’-5’ processive enzyme, and at the 3’ end of the hybrid molecules generated after the second cycle, the DNA is double-stranded and therefore not a target for the exonuclease. After treatment of the diluted first stage sample with Exo-SAP, 3 microliters of treated sample was transferred to a second PCR reaction, as was performed for the TAS (standard) PCR method. Here, the sequencing adapter and sample-specific unique barcode were incorporated using PCR. In the TAS approach, only 8 cycles were used to incorporate sequencing adapters, but for the PEX PCR method, 28 amplification cycles were performed since this reaction is intended to amplify targets and incorporate sequencing adapters.

Reactions in which the standard 806R primer was replaced with the 806R primer containing 5-nitroindole substitutions were not found to amplify well under standard conditions (TAS PCR), and weak or no yield was generated. However, the nitroindole substituted primer did work effectively in reactions employing the PEX PCR method protocol, and this may be related to relatively weak template-primer interactions associated with the “universal base” substitutions.

### Determination of optimum PEX PCR method annealing temperature

**Fig 3:**
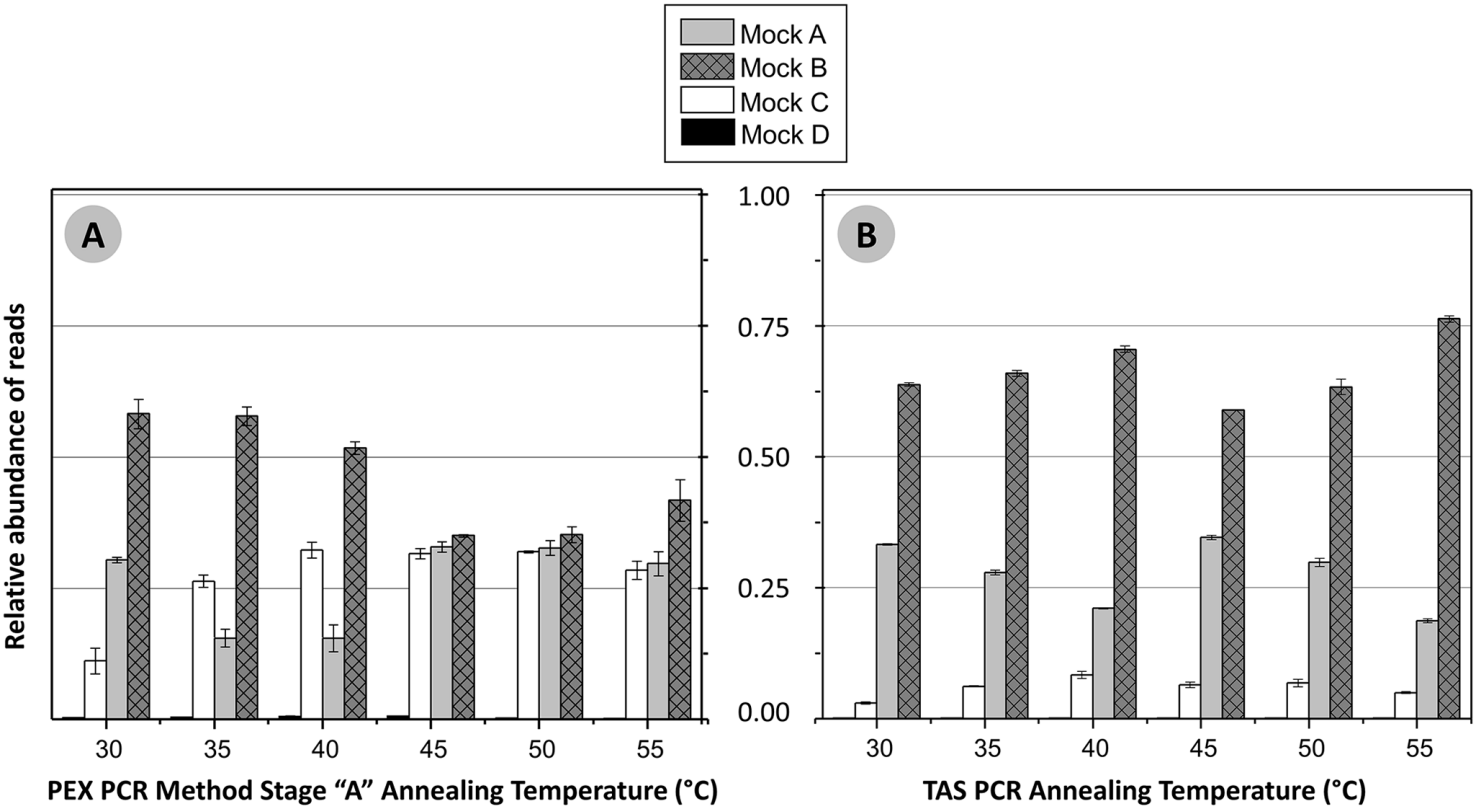
Temperature gradient analysis of the PEX PCR and TAS methods using mock community DNA. The relative abundance of reads mapping to the each of the four target templates (Mock A, Mock B, Mock C and Mock D) is shown for each temperature. The error bars represent standard deviation associated with two to four replicates per sample. **(A)** Results from PEX PCR and **(B)** Results from TAS PCR.

To determine the optimum temperature for the PEX PCR protocol with primer set 2 (Table 1), a temperature gradient was performed with stage “A” reactions containing the mock community DNA. At each temperature (30°C–55°C, 5°C increments), two cycles of 20 minute annealing and elongation were performed. After exonuclease treatment (stage “E”), each pool of fragments was amplified with the stage “B” primers, containing a unique barcode. Each condition was performed in quadruplicate, and a minimum of 8,493 sequences per sample were generated (Ion Torrent PGM). The distribution of reads among the four templates at each temperature is shown in Fig 3A. Ideal scores were calculated for all four templates (IS4), for all templates excepting the two mismatch template (IS3) and for only perfectly matching templates (IS2), and compared to results from TAS PCR (below) (S4 File). Samples processed with the PEX PCR method had significantly lower IS scores for all temperatures when considering 3 or 4 templates (IS3 and IS4; S4 File). When considering only the two perfect match templates (Mock A and Mock B), IS2 scores were significantly lower for all PEX PCR reaction temperatures except 30°C and 40°C (IS2; S4 File). Overall, the two mismatch template (Mock D) was not well amplified at any temperature, although slightly higher amplification (still below 1% of total reads) was observed in PEX PCR reactions with 40°C and 45°C annealing temperatures. Reactions using 45°C and 50°C reaction temperatures during the first stage of the PEX PCR method were capable of nearly even amplification all templates except Mock D (Fig 3A; S4 File).

For comparison with TAS (standard) PCR, a modified stage 1 PCR amplification was performed on mock community DNA to best approximate cycling conditions in the PEX PCR method. Briefly, a two-step PCR cycle was used, and a single annealing temperature was held for two minutes (*i*.*e*., 95°C—30”, AT—2’; repeated 28 times). The distribution of reads (minimum of 5,902 sequences per replicate) among the four templates at each temperature is shown in Fig 3B and S4 File. The TAS protocol preferentially amplified the template perfectly matching the reverse primer with the highest Tm (Mock B), and this varied little with annealing temperature. TAS PCR poorly amplified templates with introduced mismatches (*i*.*e*. Mock C and D).

**Fig 4:**
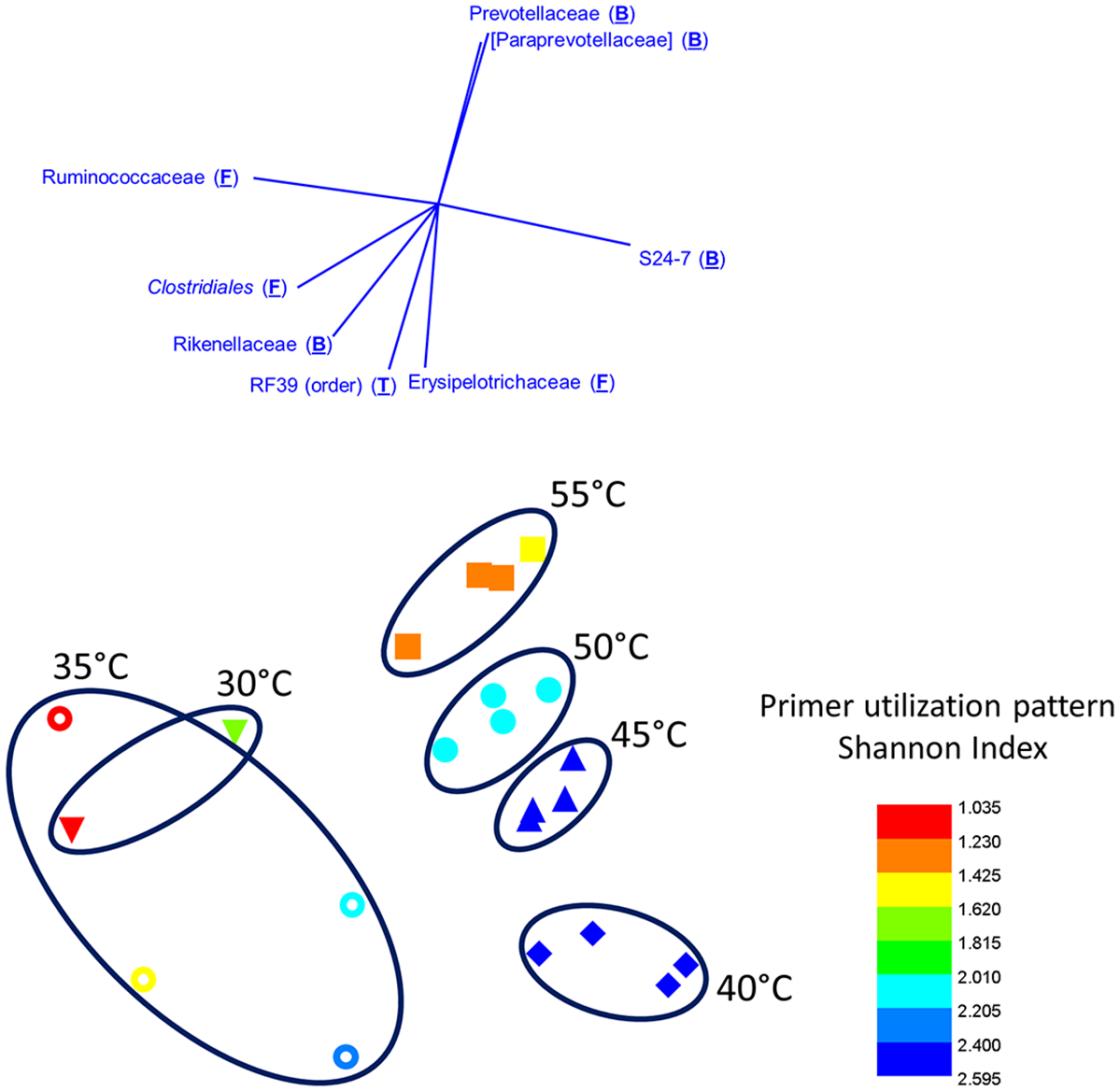
Effect of PEX PCR Stage “A” annealing temperature on observed microbial community structure and primer utilization patterns. Non-metric multidimensional scaling (NMDS) plot of fecal microbiome, performed at the taxonomic level of family and based on Bray-Curtis similarity (2D stress = 0.05). Samples were rarefied to 1,250 sequences per sample and no transformation was applied. The analysis is based on a single genomic DNA sample, with PEX PCR stage “A” annealing performed at 30°C (down-facing triangles), 35°C (open circles), 40°C (diamonds), 45°C (up-facing triangles), 50°C (closed circles) and 55°C (squares). Symbols are color-coded by the diversity (Shannon Index) of reverse primers (*i*.*e*. 806R) utilized in annealing and elongation during stage “A” of PEX PCR. Maximum possible Shannon index for 18 primers in the primer pool is 2.89. Vectors indicate taxa with Pearson correlation of >0.8 with MDS1 and MDS2 axes.

A temperature gradient was also performed using the PEX PCR method, with a single gDNA sample extracted from mammalian feces (“Chin”) used as the template (Fig 4). Data were processed as described above, and analyses were performed at the taxonomic level of family. Poor amplification of environmental gDNA was found with PEX PCR method stage A annealing temperatures below 40°C, and replicates were more variable than at higher temperatures. The observed microbial community was similar at all temperatures above 40°C (>80% Bray-Curtis similarity), with temperatures 45°C–55°C most similar (>90% Bray-Curtis similarity). Nonetheless, the observed microbial community was significantly different when the “Chin” sample was processed using different PEX PCR method stage “A” annealing temperatures (ANOSIM, R = 0.746, p-value<0.0002) due to annealing temperature-associated shifts in the relative abundance of individual taxa. For example, the relative abundance of sequences from bacteria of the family Prevotellaceae was correlated with stage “A” annealing temperature (Fig 4). Based on results from the mock community, and analysis of “Chin” gDNA, a reaction temperature of 45°C was chosen for the first stage of the PEX PCR method in subsequent analyses using the CS1_515F and CS2_806R primer set.

### Analysis of a mock community using standard and PEX PCR method protocols

**Fig 5:**
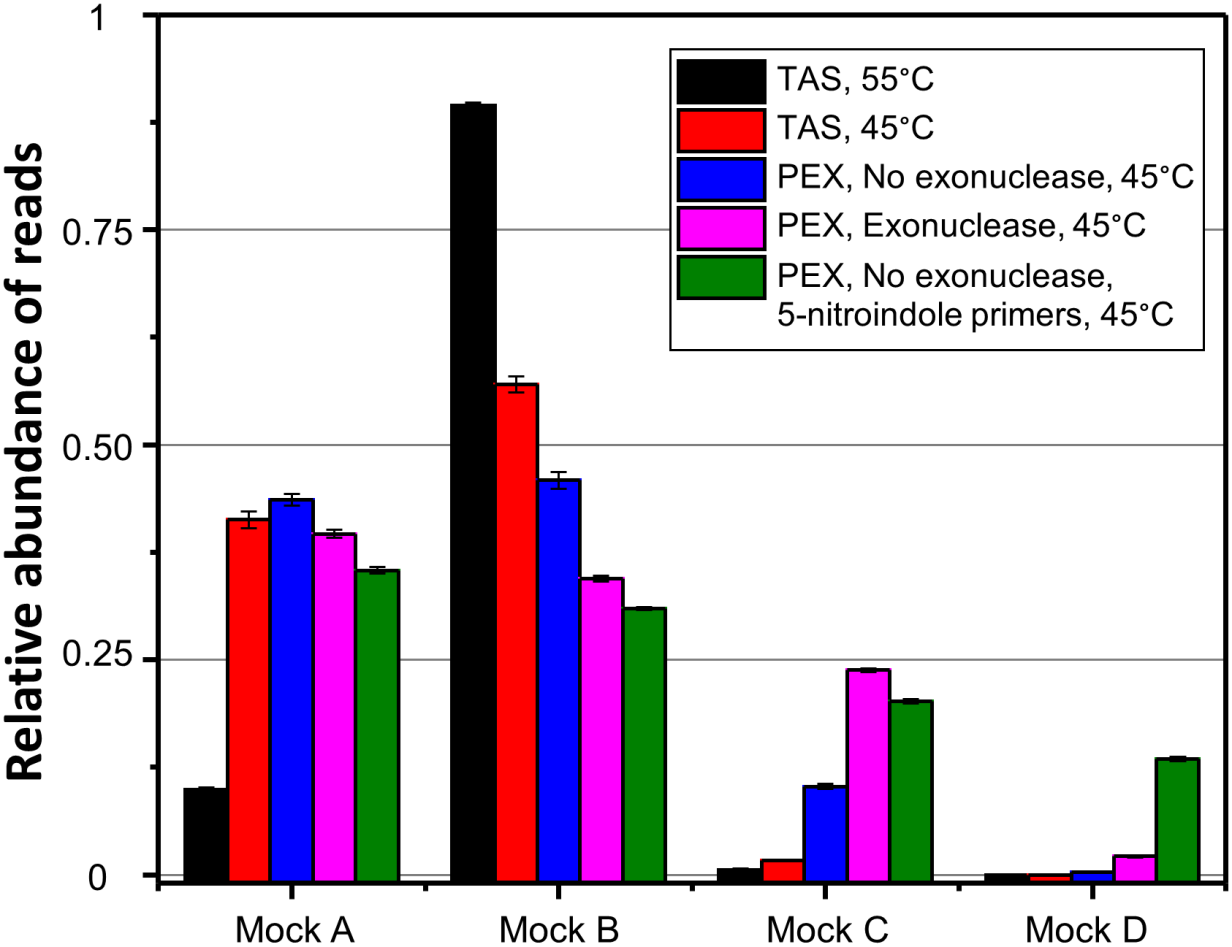
Relative abundance of mock DNA templates observed in sequencing of TAS and PEX PCR method reactions. The error bars represent standard deviation associated with two replicates per sample.

A series of tests with mock community DNA were performed to determine if the PEX PCR method protocol improved the evenness of recovery of target templates with varying sequences at primer sites. Mock community templates were amplified at 45°C and 55°C annealing temperatures using TAS PCR, at 45°C using the PEX PCR method with and without exonuclease treatment, and at 45°C using the PEX PCR method without exonuclease, but with the 806R-NI primers. Sequencing for these amplicons was performed using an Illumina MiSeq. The results of these analyses, similar to that of prior sequencing on the Ion Torrent PGM, revealed that TAS PCR, at 55°C annealing temperature, grossly distorted the underlying ratio of templates in the mock community (Table 2, Test 1; Fig 5). By lowering the annealing temperature to 45°C, the reaction could more evenly amplify perfectly matching templates (*i*.*e*. Mock A and Mock B), but still poorly amplified both DNA fragments containing mismatches introduced at the 3’ end of the priming sites.

**Table 2:** Amplification and sequence analyses of mock community DNA.

	Conditions Employed				Average percentage of reads mapping to references				Ideal Scores (IS)		
Test	Method^#^	AT^#^	Exo^#^	Reverse Primer	Mock A	Mock B	Mock C	Mock D	4 targets	3 targets	2 targets
1	TAS	45°C	No	806R	41.27	57.03	1.70	0.00	97*	63*	16*
	TAS	55°C	No	806R	9.91	89.47	0.61	0.01	129	112	80
2	TAS	45°C	No	806R	41.27	57.03	1.70	0.00	97	63	16
	PEX	45°C	No	806R	43.60	45.86	10.27	0.27	79*	46*	3*
3	PEX	45°C	No	806R	43.60	45.86	10.27	0.27	79	46	3^
	PEX	45°C	Yes	806R	39.66	34.40	23.79	2.16	48*	18*	7
4	PEX	45°C	Yes	806R	39.66	34.40	23.79	2.16	48	18*	7
	PEX	45°C	No	806R-NI	35.39	30.96	20.19	13.46	33*	20	7^NS^

^#^ TAS PCR = Targeted amplicon sequencing (standard PCR approach); PEX = Polymerase exonuclease PCR method; Exo = Exonuclease treatment

* Significant decrease relative to alternate method; p<0.01, two-tailed TTEST (unequal variance)

^^^ Significant decrease relative to alternate method; p<0.05, two-tailed TTEST (unequal variance)

^NS^ = Not significant, two-tailed TTEST (unequal variance)

DNA fragments containing a single introduced mismatch at the forward primer site (Mock C) were amplified more effectively using the PEX PCR method, even without exonuclease (Table 2, Test 2). However, this fragment was most effectively targeted when the exonuclease step was included in the PEX PCR reaction (Table 2, Test 3). The two mismatch fragment (Mock D) was not amplified effectively even in the PEX PCR reaction with exonuclease, and less than 3% of the reads were derived from the two mismatch fragment (Mock D). The use of 5-nitroindole substitutions in the 806R primer pool, decreasing the level of degeneracy of the reverse primer pool from 18X to 2X, yielded improved amplification of the two mismatch template (Mock D) and slightly higher than 13% of the reads were derived from this template (Table 2, Test 4). Overall, the “ideal score”, an estimate of how close to the true ratio of the input template the observed distribution is, demonstrated substantial and significant improvement with (i) a decrease in annealing temperature to 45°C, (ii) the use of the PEX PCR method, and (iii) substitution of 5-nitroindole for 3-fold degenerate positions in the 806R primer. Removal of exonuclease treatment decreased the evenness of the amplification reaction.

### Analysis of environmental DNA using standard and PEX PCR method protocols

**Fig 6:**
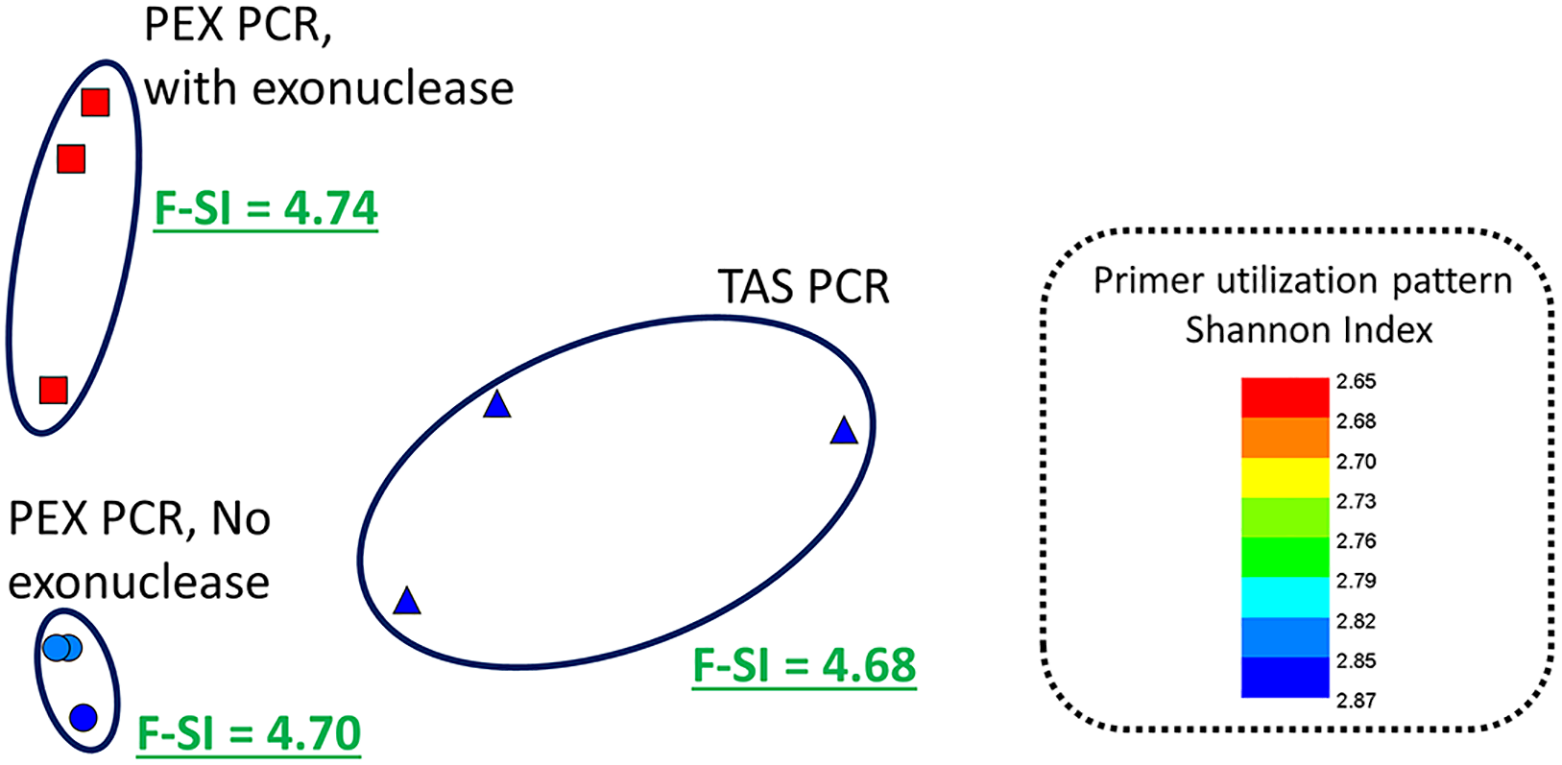
Effect of PEX PCR and exonuclease treatment on observed microbial community structure and primer utilization patterns. Non-metric multidimensional scaling (NMDS) plot of lake sediment microbiome, performed at the taxonomic level of family and based on Bray-Curtis similarity (2D stress = 0.02). Samples were rarefied to 35,500 sequences per sample and no transformation was applied. All reactions were performed with an annealing temperature of 45°C, using PEX PCR with exonuclease (squares), PEX PCR without exonuclease (circles), and TAS PCR (triangles). Symbols are color-coded by the diversity (Shannon Index) of reverse primers (*i*.*e*. 806R) detected in the sequences. Maximum possible Shannon index for 18 primers in the primer pool is 2.89. Small, but significant differences in the observed family-level Shannon index (F-SI) were observed between PEX PCRs (with and without exonuclease treatment) and TAS PCRs using a two-tailed ttest (p<0.05).

Genomic DNA extracted from sediment from Lake Huron was recovered and analyzed using the TAS PCR approach, PEX PCR method and PEX PCR method without exonuclease (Fig 6). In addition, the effect of primers containing 5-nitroindole substitutions was also examined (S2 Fig). All reactions were performed with an annealing temperature of 45°C and sequencing was performed on the Illumina MiSeq instrument. Bacterial SSU rRNA amplicon sequences were clustered and analyzed at the taxonomic level of family. A significant effect of method (TAS vs PEX PCR method) was observed (ANOSIM, R = 0.778, p<0.02), as well as a significant effect of reverse primer pool (806R vs 806R-NI; R = 0.763, p<0.0003). The observed sediment sample community differed when processed using the PEX PCR method with or without exonuclease (across both regular and nitroindole primers), though analysis of similarity was not significant (R = 0.178; p<0.07). Calculation of Shannon indices for the “Sed” sample indicated that the family diversity observed in PEX PCR amplification was slightly, but significantly, higher than that observed for TAS amplification (Fig 6). Replicates of lake sediment microbial communities in analyses with the 806R-NI primer showed poorer amplification and much greater variation than replicates from the same sample with the standard 806R primer pool (S2 Fig).

### Analysis of primer utilization patterns

The PEX PCR method allows for direct examination of which primers from a primer pool are involved in annealing and polymerase extension during the first cycles of amplification reactions, when ‘natural’ primer-template interactions occur. In standard PCRs, the primer sites are not informative, because during many cycles of amplification, different primers from a primer pool amplify target molecules during ‘artificial’ primer-template amplification cycles (*e*.*g*. cycles 3 and above; Fig 2B). In the PEX PCR method, the CS1 and CS2 portions of the primer are used for exponential amplification, and the sequence of the initial primer from the primer pool annealing to the genomic DNA template is preserved during subsequent amplification cycles in Stage “B” (Fig 1). The primer utilization pattern was initially examined in the analysis of mock community DNA across a temperature gradient (Fig 4; S3 Fig). All samples amplified with the TAS method had highly similar primer utilization profiles (>90% Bray-Curtis similarity), and these profiles showed that all primers within the reverse primer pool were involved in amplification (S3 Fig). This is an indication of the signal “scrambling” that occurs in standard PCR; when ‘artificial’ primer-template interactions dominate, primers with mismatches to amplicons can still readily anneal to these targets. A strong effect of annealing temperature on primer utilization pattern in PEX PCR-amplified samples was observed. At the highest annealing temperature (55°C), a strong skewing towards the primer with the lowest Tm was observed (S3 Fig). In addition, at this 55°C annealing temperature, the primer with the highest Tm was also a large contributor to annealing and extension. Since the mock community is composed of two templates perfectly matching the lowest Tm primer, one template with a single mismatch with the lowest Tm primer, and one template with no mismatches with the highest Tm primer (S2 File), this indicates that at the highest annealing temperatures, perfect match or few mismatch interactions are heavily favored. When the annealing temperature in the PEX PCR method was dropped to 45°C, a more even distribution of primer utilization was observed, and the primer with the lowest Tm was less strongly favored. To rapidly analyze primer utilization patterns, a Shannon Index (SI) was calculated for the distribution of reverse primers (18 possible combinations; maximum SI = 2.89). Between annealing temperatures of 55° to 40°C, a dramatic increase in the diversity of primers used for amplification was observed (Fig 4); below 40°C, an inconsistent signal was obtained (Fig 4).

To examine this phenomenon more closely, the primer utilization patterns were examined for each of the four separate templates within the mock community, using both the TAS and PEX PCR methods (S4 Fig). For TAS amplification, annealing temperature did not substantially alter primer utilization patterns, though each target had different patterns (too few sequences were recovered for template Mock D for analysis). For PEX PCR amplification, both temperature and template type altered primer utilization patterns. At high annealing temperatures, the lowest Tm primer was predominantly associated with amplification of all targets except Mock B, although the highest Tm primer contributed substantially. For the Mock B templates, the highest Tm primers were the most strongly associated with annealing and elongation. When using the PEX PCR method at 45°C, a broader array of primers were involved in annealing and elongation of Mock A, Mock C, and Mock D targets. A wide array of primers were also involved in annealing and elongation of the Mock B template, but here the primer with the highest Tm (perfectly matching the reverse primer site) was abundantly utilized.

When examining the primer utilization patterns for the “Sed” sample (lake sediment), a very diverse utilization pattern was observed for the PEX PCR method (Fig 6). This finding is consistent with the high microbial diversity in the “Sed” sample, even when compared to the fecal microbiome sample. The TAS method showed the most diverse pattern, consistent with the hypothesis that standard PCR “scrambles” the signal of primer utilization. When the same sample was processed using the PEX PCR method, but without exonuclease treatment, the resulting primer utilization pattern was most similar to that of the sample processed with the PEX PCR method, but a more even distribution of primer utilization (*i*.*e*. higher primer Shannon index) was observed, similar to the TAS PCR sample (Fig 6). This observation is an indication that carryover of primers from stage “A” to the stage “B” of the PEX PCR method is responsible for distortion during PCR amplification. Samples processed with the primer pool containing 5-nitroindole substitutions had a strong selection for a single primer from the pool (S2 Fig). This represents a preference for specific bases when the polymerase uses a 5-nitroindole base as a template for copying. In reactions with primers containing 5-nitroindole substitutions, recovery of an additional 14 primer variants was observed (S2 Fig), though at a very low level (<0.1%).

## Discussion

We describe here a novel technique for amplifying DNA to reduce the negative effects of mismatches between primer and template on the efficiency of amplification of target templates. This method is an extremely general and simple modification to the PCR reaction, and involves three enzymatic steps, including a polymerase-mediated linear copying of genomic DNA templates, an exonuclease digestion of primers, and a final PCR amplification using primers targeting target-independent linker sequences. As shown in this manuscript, this approach allows the exponential amplification of the target of interest to be performed with primers that have no mismatches with any templates in the reaction. This significantly reduces bias associated with mismatches and degenerate primers that can accumulate during PCR by limiting primer-template interactions to two cycles only.

The objectives of this study were to: (i) demonstrate that the first and second stages of PCR could be separated, and still generate reliable amplification; (ii) determine if degeneracies in the primer pools led to obvious distortion of the observed microbial community, and if the PEX PCR technique could be used to circumvent or reduce such distortion, (iii) develop a robust workflow for this approach to be implemented for any primer set or sets, and (iv) identify applications to which this approach is best suited. The results herein demonstrate that indeed the two defined types of interactions within PCR (*i*.*e*. natural and artificial interactions) can and should be separated when degenerate primers are used or when mismatches with the template are anticipated. The stages should be separated because genomic DNA template-primer interactions have the greatest potential for bias due to mismatches derived from true mismatches in the gDNA and from degeneracies in the primer pool. Our analysis of an artificially synthesized mock community demonstrates the strong potential for a degenerate primer pool of oligonucleotides of varying melting temperatures to preferentially select templates based on sequence variations in the primer site. Our strategy limits the gDNA template-primer interaction to two cycles, with all subsequent amplification cycles employing non-degenerate, non-template interactions. Furthermore, because only two cycles are utilized during the first stage of gDNA-primer interactions, unusual annealing and elongation conditions can be utilized. In this study, we employed long annealing times (20 minutes) at low annealing temperatures to allow for adequate time for the polymerase to bind target sites and elongate without raising the reaction temperature. Such reaction conditions are likely more tolerant of low primer annealing efficiency of primer-template pairings with mismatches. In a systematic study of primer-template interactions, Wu et al. [31] reported that single mismatches occurring in the last 3–4 positions from the 3’ end of the primer yielded minimal or no primer extension. We observe here that 3’ mismatches can be overcome using the PEX PCR method, and that primers with 3 or 4 mismatches can still anneal with genomic DNA targets and yield polymerase extension. This was revealed through an analysis of primer utilization patterns in mock community analyses. We note that such an analysis cannot be performed using standard PCR approaches, and requires the PEX PCR method.

Furthermore, we demonstrate through the comparison of PEX PCR method with and without exonuclease that the primers targeting the source gDNA (or mock DNA) do contribute to the distortion during later cycles of PCR even in the presence of high concentrations of the second stage PCR primers. The complete removal of unincorporated primers from the first stage reaction, although desirable, was found to be difficult. Even after dilution, exonuclease digestion, and lowering of the primer concentration during the first two cycles, some limited amount of first stage primer may be propagated to the second stage PCR. Despite this, treatment with exonuclease significantly reduces the impact of primer carry-over, and is an essential part of the PEX PCR methodology. In analyses of both mock community and environmental gDNA, exonuclease treatment significantly alters the observed microbial community. It is possible that in place of exonuclease treatment, blocking oligonucleotides of the inverse complement of the forward and reverse template-specific primers (*e*.*g*. inverse complement of 515F and 806R primers without CS1 and CS2 linkers) could be added to the second PCR stage of the PEX PCR reaction to prevent gDNA template-primer interactions.

The PEX PCR method also provides a novel and robust mechanism to explore primer-template interactions in analyses of complex gDNA samples. The PEX PCR method preserves the sequence of the primer annealing to the gDNA template during the first two cycles of the reaction, and these can be bioinformatically interrogated to determine which primers within a degenerate pool are truly involved in annealing and extension. We observed that at high annealing temperatures, perfect match annealing is favored, and a lower diversity of the primers in the pool were utilized. This appears to be detrimental for the amplification reaction, as perfectly matching primers are present at a low overall abundance in a heavily degenerate primer pool. At lower annealing temperatures, a broader spectrum of primers, containing mismatches with the template, are involved in annealing and elongation. This appears to be beneficial, as analyses of the mock community under lower annealing conditions, generated better representations of the true underlying distribution of mock DNAs. Primers with 0–4 mismatches with various templates were observed to anneal and allow for polymerase extension. When 3’ mismatches were introduced into mock DNA templates, the primer utilization distribution shifted towards primers with 1 or 2 total mismatches to the template. Therefore, the heavy degeneracy of primer sets may not be beneficial when using the PEX PCR method. Instead, it may be appropriate to select “intermediate” primers that have at most 1 or 2 mismatches to all potential priming sites, with the assumption that every variant does not require a unique primer to be targeted. Further research is needed to determine the best combination of primer degeneracy and annealing temperatures for other, more degenerate targets such as microbial functional genes. Such a strategy may enable a direct PCR-based method for analysis of a single copy gene present in all microorganisms for the purpose of community structure analyses. We further note that additional strategies may be required to allow the PEX PCR method to work effectively at lower annealing temperatures (*e*.*g*. <45°C), such as the introduction of single-stranded DNA binding protein into the amplification master mixes.

The PEX PCR method is recommended for: (i) any PCR reaction in which a degenerate primer pool is used; (ii) any PCR reaction in which a non-degenerate primer is used but where DNA template variability at the priming site is possible; (iii) reactions in which high-level degeneracy may be utilized to target all known variants of a gene; and (iv) when multiple primer pairs are to be utilized simultaneously. We show here that the method can be used to amplify and sequence templates with mismatches at the 3’ end of the primer site, which have been shown to be highly destabilizing in PCR [31,32]. Crosby and Criddle [33] previously employed a strategy using hybridization capture followed by random-primed amplification and sequencing to target DNA-directed RNA polymerase genes (*rpoC*). The PEX PCR method may be adaptable to a direct PCR amplification of *rpoC* genes from all microorganisms within a single amplification reaction. This would preserve the original relative abundance found in the template DNA, and provide a direct proxy for relative abundance of organisms in the sample. This is unlike amplification and sequencing of rRNA genes, as performed in this study, since a wide range of gene copies of rRNA operons are found across the domains Bacteria and Archaea [34]. We do not yet know if the level of degeneracy at conserved regions of the *rpoC* (or other similar) gene is likely to be a major impediment during the first two cycles of stage “A” of the PEX PCR method, but this is a clear next step in the development of this technique. If primer dimerization in reactions with extremely degenerate primer pools is observed, purification of stage “A” components using a size-selection protocol (*e*.*g*. AMPure beads) instead of exonuclease will reduce the transfer of primer dimers which are insensitive to single-strand exonuclease activity.

The PEX PCR method may also find wide-spread application for quantitative PCRs in which degenerate primers are employed. Quantitative PCRs could be performed by initially processing DNA samples prepared using stage “A” of the PEX PCR method. Subsequently, qPCR would be performed using primers targeting linker sequences instead of template-specific primers. This could potentially greatly increase qPCR efficiency and target range for broad-target degenerate primers common to environmental microbiology, and avoid problems deriving from differential amplification efficiencies for different targets. A similar approach has in fact been developed, with the aim to reduce the impact of bacterial DNA contamination in qPCR reactions [35].

Finally, we note that this method has conceptual similarities to a study previously performed by Crosby and Criddle [33], in which linker sequences connected to random primers were used to amplify functional genes that were captured using hybridization probes. In that study, two cycles of annealing and elongation were used for labeling, with subsequent amplification. In addition, Illumina has developed a target-capture approach in which two primers with linkers either at the 5’ or 3’ ends are allowed to anneal to a single strand of template DNA (*i*.*e*. TruSeq Amplicon). After polymerase extension, ligation is used to link the elongated fragment to the 3’ terminal primer with a 3’ flanking linker. Subsequently, PCR amplification using the linker sequences is used to prepare fragments for sequencing.

## Supporting Information

S1 Fig Sequencing workflow using targeted-amplicon sequencing (TAS) (A) and the polymerase-exonuclease-PCR (PEX PCR) method (B). The TAS workflow consists of two PCR stages in which template-specific primers containing 5’ linker sequences are used to amplify from template DNA. Subsequently, an aliquot of the first PCR is transferred to a second reaction for amplification with primers containing NGS sequencing adapters and a sample-specific barcode. In the PEX PCR method, a modified workflow is used; the first stage reaction is truncated after 2 cycles, primers are removed using exonuclease digestion, and the exonuclease-treated reaction mixture is subsequently PCR-amplified with primers containing sequencing adapters and barcodes. AT = annealing temperature; ET = Elongation time.
(TIF)

S2 Fig Effect of 5-nitroindole substitution and amplification strategy on observed microbial community structure and primer utilization patterns. **(A)** Non-metric multidimensional scaling (NMDS) plot of lake sediment microbiome, performed at the taxonomic level of family and based on Bray-Curtis similarity (2D stress = 0.02). Samples were rarefied to 4,750 sequences per sample and no transformation was applied. Symbols are color-coded by the diversity (Shannon Index) of reverse primers (*i*.*e*. 806R) detected in the sequences. Maximum possible Shannon index for 18 primers in the primer pool is 2.89. Reactions in which the 806R primer with 5-nitroindole (806R-NI) substitutions was used were less reproducible. **(B)** Group-average dendogram of observed lake sediment microbial community structure from a single sample as amplification method is altered. gDNA was PCR amplified using the standard TAS reaction and with PEX PCR reactions with and without exonuclease and with and without primers containing 5-nitroindole substitutions. Bray-Curtis similarity scores were generated based on family-level taxonomic classification, generated as described in the text. Data were standardized but not transformed. Clusters containing all three replicates from a single treatment are indicated by a symbol at the node. **(C)** Dendogram and heatmap of reverse (806R) primer utilization patterns for the same samples. Bray-Curtis similarity was generated based on standardized abundance of each of 18 primer variants present in the reverse primer pool. The heatmap indicates relative abundance of each primer variant for each sample, with primers ordered by increasing theoretical Tm. A separate column (at the very bottom) indicates the relative abundance of variants potentially present when 5-nitroindole primers are used.
(TIF)

S3 Fig Effect of method and annealing temperature on primer utilization patterns in mock DNA. Dendogram and heatmap of reverse primer utilization patterns for the mock community analyzed using the TAS and PEX PCR methods, at temperatures from 30°-55°C. Bray-Curtis similarity was generated based on standardized abundance of each of 18 primer variants present in the reverse primer pool. The heatmap indicates relative abundance of each primer variant for each sample, with primers ordered by increasing theoretical Tm. All reactions using the TAS method clustered together (node indicated by a blue circle). Underlined samples are analyzed at the individual template level in S4 Fig.
(TIF)

S4 Fig Effect of method and annealing temperature on primer utilization patterns for each mock template. Dendogram and heatmap of reverse primer utilization patterns for the mock community analyzed using the TAS and PEX PCR methods, at temperatures of 45° and 55°C. Bray-Curtis similarity was generated based on standardized abundance of each of 18 primer variants present in the reverse primer pool. The heatmap indicates relative abundance of each primer variant for each template within the mock community DNA pool, with primers ordered by increasing theoretical Tm.
(TIF)

S5 Fig Effect of annealing temperature on observed microbial community structure and primer utilization patterns. **(A)** Group-average dendogram of observed mammalian fecal microbial community structure from a single sample (“Chin”) as PEX PCR stage 1 annealing temperature is altered. Labeled nodes indicate grouping of replicates from a single annealing temperature (* indicates a single replicate from 55°C is included). Bray-Curtis similarity scores were generated based on family-level biological data, generated as described in the text. Data were standardized but not transformed. **(B)** Dendogram and heatmap of reverse primer utilization patterns for the same samples. Bray-Curtis similarity was generated based on standardized abundance of each of 18 primer variants present in the reverse primer pool. The heatmap (0–75%) indicates the relative abundance of each primer variant for each sample, with primers ordered by increasing theoretical Tm.
(TIF)

S1 File Sequences of artificial DNA fragments (PDF)

S2 File Primer sites and mismatches with mock double-stranded DNA templates (PDF)

S3 File List of FASTQ filenames and associated sample preparation details. (PDF)

S4 File Temperature gradient tests of PEX PCR and TAS methods using mock DNA at varying annealing temperatures. (PDF)
