# Supplementary figures and images for: Deconstructing the Polymerase Chain Reaction: Understanding and Correcting Bias Associated with Primer Degeneracies and Primer-Template Mismatches
Source: PLoS One. 2015 May 21;10(5):e0128122. doi: 10.1371/journal.pone.0128122 (PMC4440812; doi:10.1371/journal.pone.0128122)

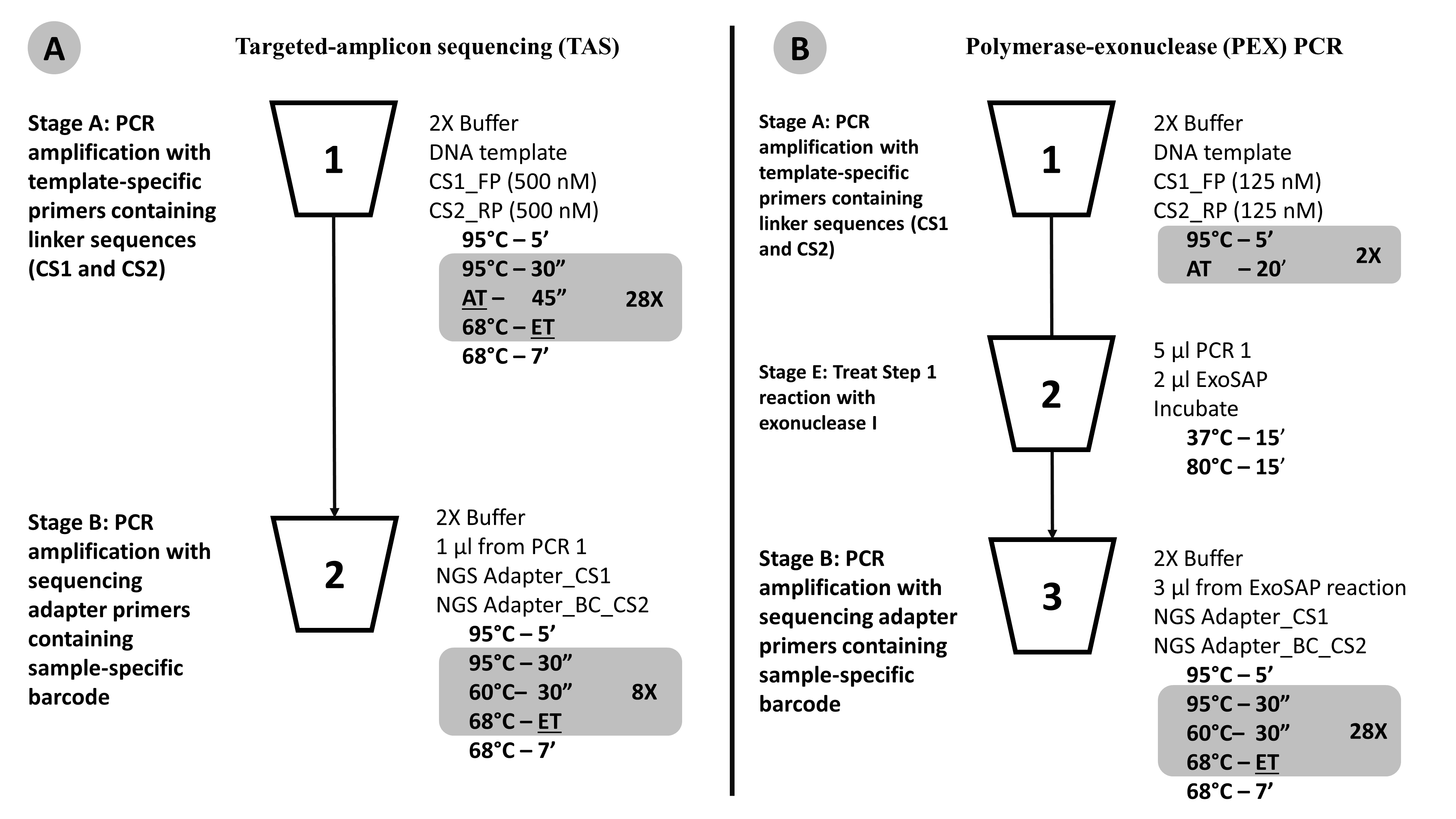

Supplement: S1 Fig — The TAS workflow consists of two PCR stages in which template-specific primers containing 5’ linker sequences are used to amplify from template DNA. Subsequently, an aliquot of the first PCR is transferred to a second reaction for amplification with primers containing NGS sequencing adapters and a sample-specific barcode. In the PEX PCR method, a modified workflow is used; the first stage reaction is truncated after 2 cycles, primers are removed using exonuclease digestion, and the exonuclease-treated reaction mixture is subsequently PCR-amplified with primers containing sequencing adapters and barcodes. AT = annealing temperature; ET = Elongation time. (TIF) [file pone.0128122.s001.TIF]

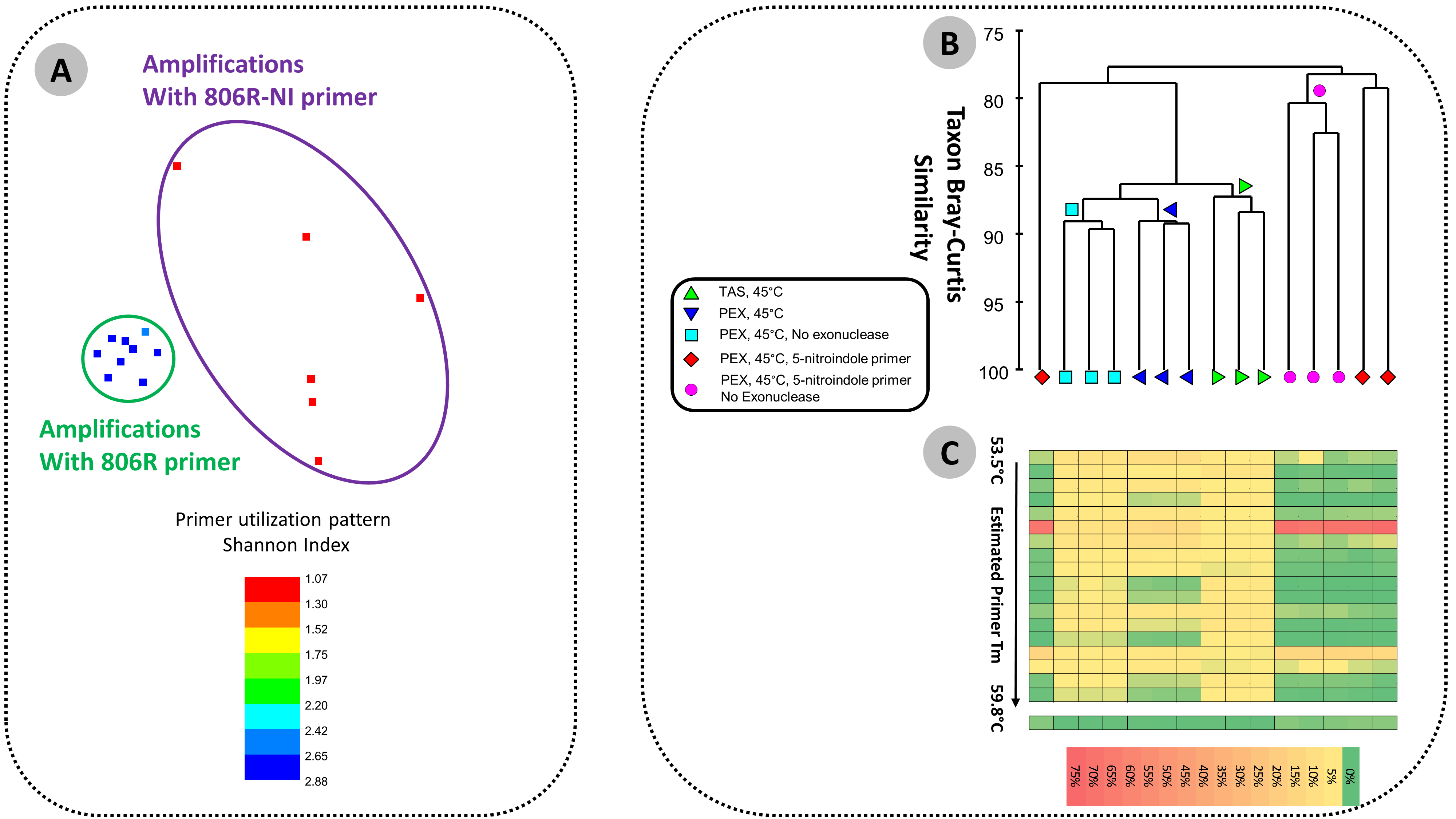

Supplement: S2 Fig — (A) Non-metric multidimensional scaling (NMDS) plot of lake sediment microbiome, performed at the taxonomic level of family and based on Bray-Curtis similarity (2D stress = 0.02). Samples were rarefied to 4,750 sequences per sample and no transformation was applied. Symbols are color-coded by the diversity (Shannon Index) of reverse primers (i.e. 806R) detected in the sequences. Maximum possible Shannon index for 18 primers in the primer pool is 2.89. Reactions in which the 806R primer with 5-nitroindole (806R-NI) substitutions was used were less reproducible. (B) Group-average dendogram of observed lake sediment microbial community structure from a single sample as amplification method is altered. gDNA was PCR amplified using the standard TAS reaction and with PEX PCR reactions with and without exonuclease and with and without primers containing 5-nitroindole substitutions. Bray-Curtis similarity scores were generated based on family-level taxonomic classification, generated as described in the text. Data were standardized but not transformed. Clusters containing all three replicates from a single treatment are indicated by a symbol at the node. (C) Dendogram and heatmap of reverse (806R) primer utilization patterns for the same samples. Bray-Curtis similarity was generated based on standardized abundance of each of 18 primer variants present in the reverse primer pool. The heatmap indicates relative abundance of each primer variant for each sample, with primers ordered by increasing theoretical Tm. A separate column (at the very bottom) indicates the relative abundance of variants potentially present when 5-nitroindole primers are used. (TIF) [file pone.0128122.s002.TIF]

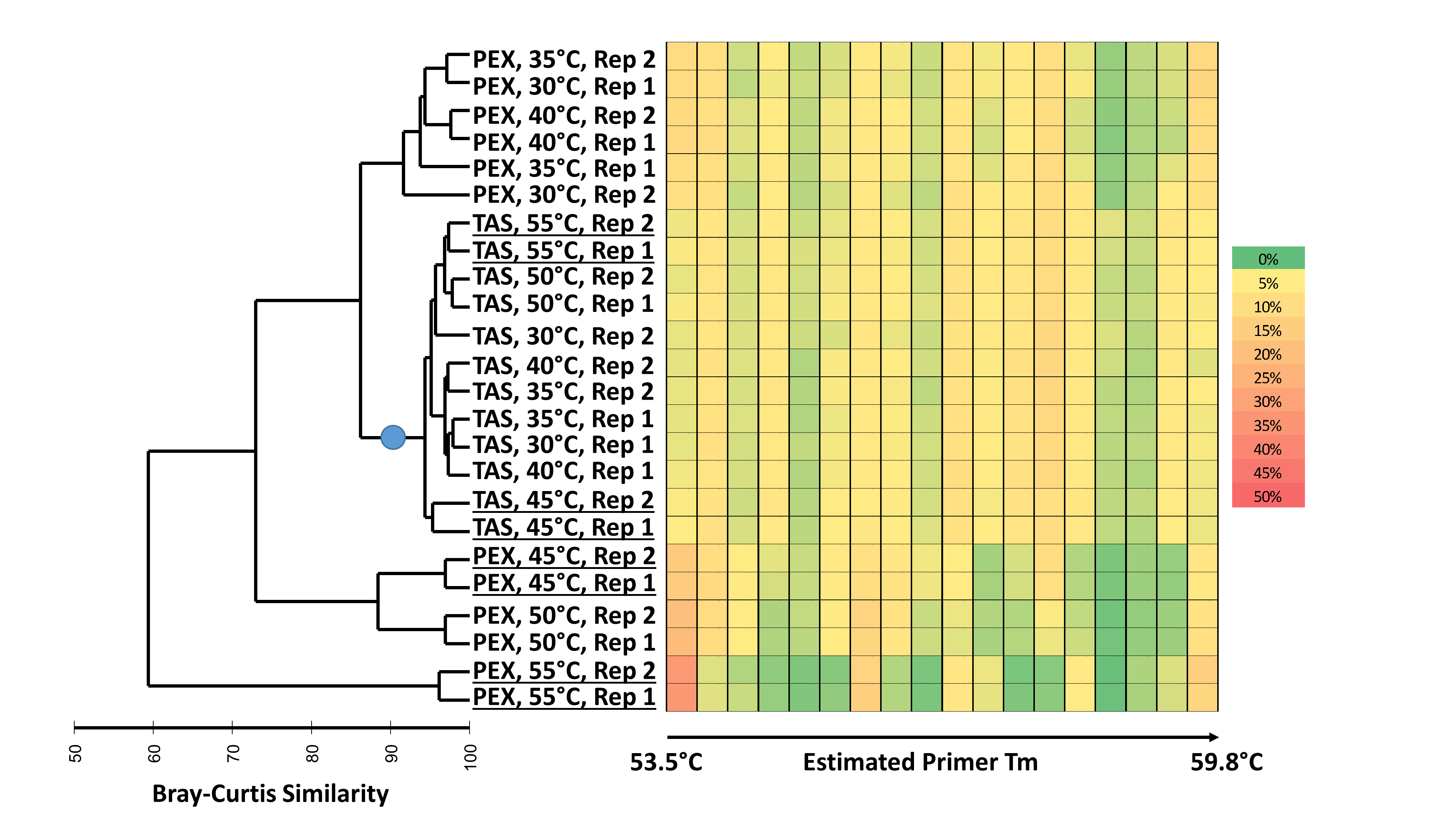

Supplement: S3 Fig — Dendogram and heatmap of reverse primer utilization patterns for the mock community analyzed using the TAS and PEX PCR methods, at temperatures from 30°-55°C. Bray-Curtis similarity was generated based on standardized abundance of each of 18 primer variants present in the reverse primer pool. The heatmap indicates relative abundance of each primer variant for each sample, with primers ordered by increasing theoretical Tm. All reactions using the TAS method clustered together (node indicated by a blue circle). Underlined samples are analyzed at the individual template level in S4 Fig. (TIF) [file pone.0128122.s003.TIF]

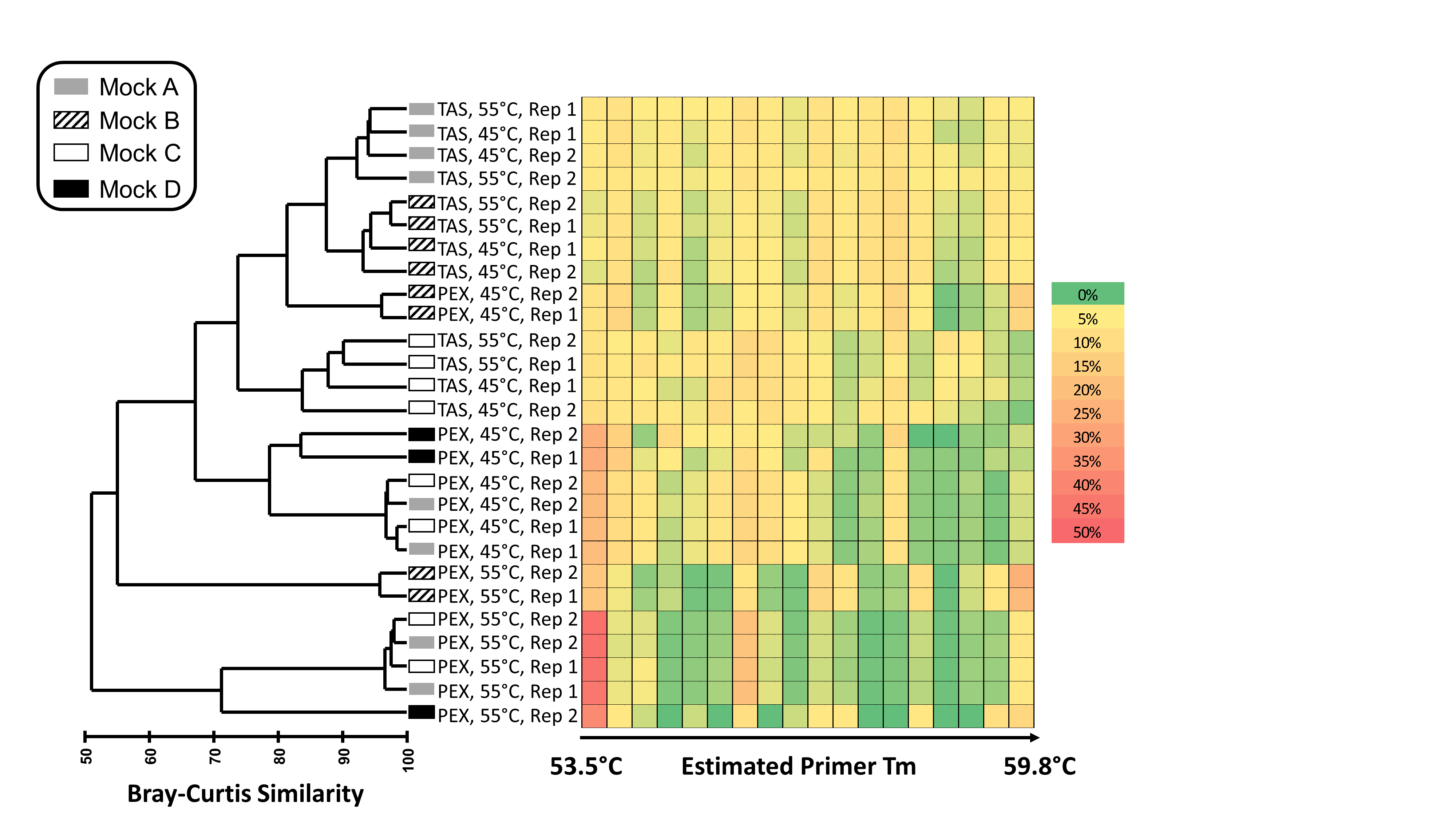

Supplement: S4 Fig — Dendogram and heatmap of reverse primer utilization patterns for the mock community analyzed using the TAS and PEX PCR methods, at temperatures of 45° and 55°C. Bray-Curtis similarity was generated based on standardized abundance of each of 18 primer variants present in the reverse primer pool. The heatmap indicates relative abundance of each primer variant for each template within the mock community DNA pool, with primers ordered by increasing theoretical Tm. (TIF) [file pone.0128122.s004.TIF]

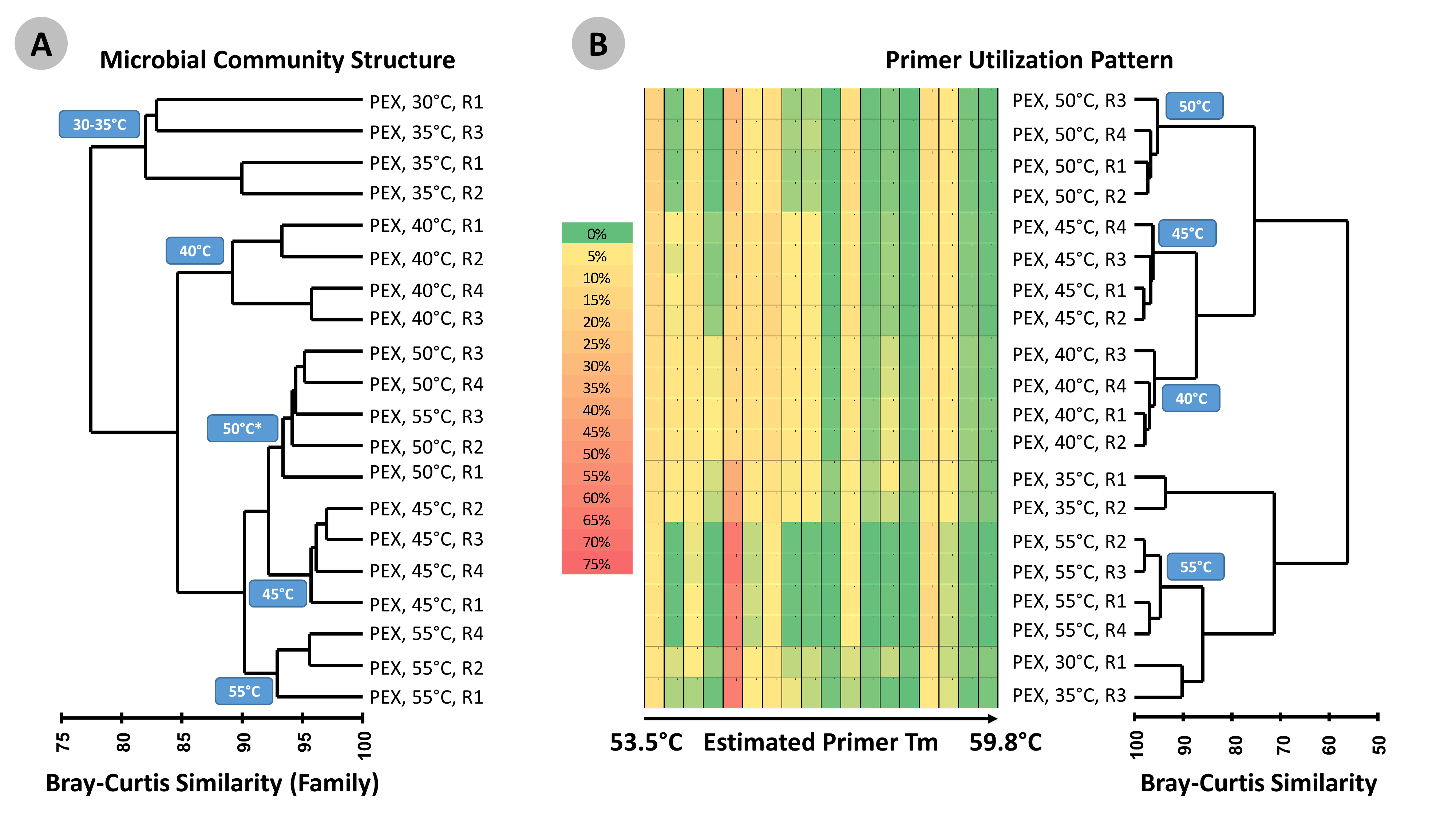

Supplement: S5 Fig — (A) Group-average dendogram of observed mammalian fecal microbial community structure from a single sample (“Chin”) as PEX PCR stage 1 annealing temperature is altered. Labeled nodes indicate grouping of replicates from a single annealing temperature (* indicates a single replicate from 55°C is included). Bray-Curtis similarity scores were generated based on family-level biological data, generated as described in the text. Data were standardized but not transformed. (B) Dendogram and heatmap of reverse primer utilization patterns for the same samples. Bray-Curtis similarity was generated based on standardized abundance of each of 18 primer variants present in the reverse primer pool. The heatmap (0–75%) indicates the relative abundance of each primer variant for each sample, with primers ordered by increasing theoretical Tm. (TIF) [file pone.0128122.s005.TIF]
