# Supplementary material for: Deconstructing the Polymerase Chain Reaction: Understanding and Correcting Bias Associated with Primer Degeneracies and Primer-Template Mismatches
Source: PLoS One. 2015 May 21;10(5):e0128122. doi: 10.1371/journal.pone.0128122 (PMC4440812; doi:10.1371/journal.pone.0128122)
Supplement: S1 File — (PDF) [file pone.0128122.s006.pdf]

Supporting Information

S1 File: Sequences of artificial DNA fragments. Artificial fragments are 492 bp gene fragments synthesized by Integrated DNA Technologies (Coralville, Iowa). The fragments are derived from the sequence of the small subunit (SSU or 16S) ribosomal RNA (rRNA) gene of *Rhodanobacter denitrificans* 2APBS1, a denitrifying Gammaproteobacteria (GenBank accession number: NR_102497.1). The sequences shown represent one strand (5'-3') of the double-stranded molecules. The sequences highlighted in green represent the target position of the 515F primer, while the light blue sequences represent the target position of 806R primer (Caporaso et al. 2011). Positions highlighted in red indicate introduced mismatches relative to the primer pool, introduced into the synthesized DNA fragments to allow for testing of effects of mismatches between template and primer. The region highlighted in purple is a 10-bp region which is diagnostic for each of the four sequences. The 10 bp are re-arranged in each region to allow for identification of the template, while maintaining overall similarity of the gene fragment. The last two gene fragments contain mismatches that are not targeted by any of the primer variants found in the standard degenerate 515F and 806R primers. The forward primer (515F) primer site is highlighted in bright green, and the reverse primer site is highlighted in cyan when matching the lowest Tm reverse primer or olive green when matching the highest Tm reverse primer.

>Mock A (FP Tm = Low / RP Tm = Low)
TAAAGCACTTTTATCAGGAGCGAAATACCACGGGTTAATACCCTATGGGGCTGACG
GTACCTGAGGAATAAGCACCGGCTAACTTCGTGCCAGCAGCCGCGGTAATACGAAG
GGTGCAAGCGTTAATCGGAATTACTGGGCGTAAAGGGTGCGTAGGCGGTTACTTAA
GTCTGTCGTGAAATCCCCGGGCTCAACCTGGGAATGGCGATGGATACTGGGTGGCTA
GAGTGTGTCAGAGGATGGTGGAATTCCCGGTGTAGCGGTGAAATGCGTAGAGATCG
GGAGGAACATCAGTGGCGAAGGCGGCCATCTGGGACAACACTGACGCTGAAGCAC
GAAAGCGTGGGGAGCAAACAGGATTAGATACCCTAGTAGTCCACGCCCTAAACGAT
GCGAACTGGATGTTGGTCTCAACTCGGAGATCAGTGTCGAAGCTAACGCGTTAAGTT
CGCCGCCTGGGGAGTACGGTCGCAAGACTGAAACTCAAAGGAA

>Mock B (FP Tm = Low / RP Tm = High)
TAAAGCACTTTTATCAGGAGCGAAATACCACGGGTTAATACCCTATGGGGCTGACG
GTACCTGAGGAATAAGCACCGGCTAACTTCGTGCCAGCAGCCGCGGTAATACGAAG
GGTGCAAGCGTTAATCGGAATTACTGGGCGTAAAGGGTGCGTAGGCGGTTACTTAA
GTCTGTCGTGAAATCCCCGGGCTCAACCTGGGAATGATAGGTAGCGCTGGGTGGCTA
GAGTGTGTCAGAGGATGGTGGAATTCCCGGTGTAGCGGTGAAATGCGTAGAGATCG
GGAGGAACATCAGTGGCGAAGGCGGCCATCTGGGACAACACTGACGCTGAAGCAC
GAAAGCGTGGGGAGCAAACAGGATTAGAAACCCGGGTAGTCCACGCCCTAAACGAT
GCGAACTGGATGTTGGTCTCAACTCGGAGATCAGTGTCGAAGCTAACGCGTTAAGTT
CGCCGCCTGGGGAGTACGGTCGCAAGACTGAAACTCAAAGGAA

>Mock C (FP Tm = Low + mismatch / RP Tm = Low)
TAAAGCACTTTTATCAGGAGCGAAATACCACGGGTTAATACCCTATGGGGCTGACG
GTACCTGAGGAATAAGCACCGGCTAACTTCGTGCCAGCAGCCGCGGTCATACGAAG
GGTGCAAGCGTTAATCGGAATTACTGGGCGTAAAGGGTGCGTAGGCGGTTACTTAA
GTCTGTCGTGAAATCCCCGGGCTCAACCTGGGAATGGACTGAAGTGCTGGGTGGCTA
GAGTGTGTCAGAGGATGGTGGAATTCCCGGTGTAGCGGTGAAATGCGTAGAGATCG
GGAGGAACATCAGTGGCGAAGGCGGCCATCTGGGACAACACTGACGCTGAAGCAC
GAAAGCGTGGGGAGCAAACAGGATTAGATACCCTAGTAGTCCACGCCCTAAACGAT
GCGAACTGGATGTTGGTCTCAACTCGGAGATCAGTGTCGAAGCTAACGCGTTAAGTT
CGCCGCCTGGGGAGTACGGTCGCAAGACTGAAACTCAAAGGAA

>Mock D (FP Tm = Low + mismatch / RP Tm = Low + mismatch)
TAAAGCACTTTTATCAGGAGCGAAATACCACGGGTTAATACCCTATGGGGCTGACG
GTACCTGAGGAATAAGCACCGGCTAACTTCGTGCCAGCAGCCGCGGTCATACGAAG
GGTGCAAGCGTTAATCGGAATTACTGGGCGTAAAGGGTGCGTAGGCGGTTACTTAA
GTCTGTCGTGAAATCCCCGGGCTCAACCTGGGAATGGGATAGCGATCTGGGTGGCTA
GAGTGTGTCAGAGGATGGTGGAATTCCCGGTGTAGCGGTGAAATGCGTAGAGATCG
GGAGGAACATCAGTGGCGAAGGCGGCCATCTGGGACAACACTGACGCTGAAGCAC
GAAAGCGTGGGGAGCAAACAGGAGTAGATACCCTAGTAGTCCACGCCCTAAACGAT
GCGAACTGGATGTTGGTCTCAACTCGGAGATCAGTGTCGAAGCTAACGCGTTAAGTT
CGCCGCCTGGGGAGTACGGTCGCAAGACTGAAACTCAAAGGAA
