# Supplementary material for: Deconstructing the Polymerase Chain Reaction: Understanding and Correcting Bias Associated with Primer Degeneracies and Primer-Template Mismatches
Source: PLoS One. 2015 May 21;10(5):e0128122. doi: 10.1371/journal.pone.0128122 (PMC4440812; doi:10.1371/journal.pone.0128122)
Supplement: S2 File — (PDF) [file pone.0128122.s007.pdf]

Supporting Information

S2 File: Primer sites and mismatches with mock double-stranded DNA templates. Degenerate positions in the primer pools are indicated with red, underlined letters. For each of the mock DNA templates (Mock A, B, C and D) number of variants with each individual primer in the pool is indicated. Additional mismatches were introduced near the 3' end of the annealing site for forward and reverse primers, as indicated by red, underlined letters for Mock C and D templates.

			Mock A	Mock B	Mock C	Mock D
			(L/L)	(L/H)	(L+1mm/L)	(L+1mm/L+1mm)
**Forward primers**	**Primer sequence**	**Tm***	GTGCCAGCAGCC GCGGTAA	GTGCCAGCAGCC GCGGTAA	GTGCCAGCAGCC GCGGT<u>C</u>A	GTGCCAGCAGCC GCGGT<u>C</u>A
**Degenerate Pool**	**GTGCCAGC<u>M</u>GCCGCGGTAA**		0	0	1	1
FP-Variant 1	GTGCCAGCAGCCGCGGTAA	68.5	0	0	1	1
FP-Variant 2	GTGCCAGCCGCCGCGGTAA	70.7	1	1	2	2
**Reverse Primers**	**Primer sequence**	**Tm***	GGACTACTAGGG TATCTAAT	GGACTACCCGGG TTTCTAAT	GGACTACTAGGG TATCTAAT	GGACTACTAGGG TATCTA<u>C</u>T
**Degenerate Pool**	**GGACTAC<u>HV</u>GGGT<u>W</u>TCTAAT**		0	0	0	1
RP-Variant 1	GGACTACTAGGGTATCTAAT	53.5	0	3	0	1
RP-Variant 2	GGACTACTAGGGTTTCTAAT	54.6	1	2	1	2
RP-Variant 3	GGACTACAAGGGTATCTAAT	54.7	1	3	1	2
RP-Variant 4	GGACTACAAGGGTTTCTAAT	55.7	2	2	2	3
RP-Variant 5	GGACTACCAGGGTATCTAAT	56.4	1	2	1	2
RP-Variant 6	GGACTACAGGGGTATCTAAT	56.4	2	3	2	3
RP-Variant 7	GGACTACTGGGGTATCTAAT	56.4	1	3	1	2
RP-Variant 8	GGACTACTCGGGTATCTAAT	56.4	1	2	1	2
RP-Variant 9	GGACTACACGGGTATCTAAT	57	2	3	2	3
RP-Variant 10	GGACTACTCGGGTTTCTAAT	57.4	2	1	2	3
RP-Variant 11	GGACTACCAGGGTTTCTAAT	57.5	2	1	2	3
RP-Variant 12	GGACTACAGGGGTTTCTAAT	57.5	3	2	3	4
RP-Variant 13	GGACTACTGGGGTTTCTAAT	57.5	2	2	2	3
RP-Variant 14	GGACTACACGGGTTTCTAAT	58	3	1	3	4
RP-Variant 15	GGACTACCGGGGTATCTAAT	58.7	2	2	2	3
RP-Variant 16	GGACTACCCGGGTATCTAAT	58.7	2	1	2	3
RP-Variant 17	GGACTACCGGGGTTTCTAAT	59.8	3	1	3	4
RP-Variant 18	GGACTACCCGGGTTTCTAAT	59.8	3	0	3	4

* Tm (°C) calculated using OligoAnalyzer3.1, assuming 125 nM primer concentration, 2 mM Mg^{2+}, and 0.2 mM dNTPs.
