# Supplementary material for: Deconstructing the Polymerase Chain Reaction: Understanding and Correcting Bias Associated with Primer Degeneracies and Primer-Template Mismatches
Source: PLoS One. 2015 May 21;10(5):e0128122. doi: 10.1371/journal.pone.0128122 (PMC4440812; doi:10.1371/journal.pone.0128122)
Supplement: S3 File — (PDF) [file pone.0128122.s008.pdf]

Supporting Information

S3 File: List of FASTQ filenames and associated sample preparation details (BioProject PRJNA262579).

Sample Name	Sequencer	Barcode / MID	DNA Template	PEP/TAS	Temp(°C)	Exo-nuclease?	Primer (806R/806_NI)
M1_1_2cy_28cy	MiSeq	TGCTACATCA	Mock	PEP	45	No	806R
M1_2_2cy_28cy	MiSeq	TGATAGAGAG	Mock	PEP	45	No	806R
M1_3_2cy_28cy	MiSeq	CTCAGCAGTG	Mock	PEP	45	No	806R
M1e_1_2cy_28cy	MiSeq	CAGCTATAGC	Mock	PEP	45	Yes	806R
M1e_2_2cy_28cy	MiSeq	GACTCATGCT	Mock	PEP	45	Yes	806R
M1e_3_2cy_28cy	MiSeq	CACATACAGT	Mock	PEP	45	Yes	806R
m1_1_45c_28_8	MiSeq	AATATGCTGC	Mock	TAS	45	No	806R
m1_2_45c_28_8	MiSeq	AGAGGTCGGA	Mock	TAS	45	No	806R
m1_3_45c_28_8	MiSeq	ATCTGTCCAT	Mock	TAS	45	No	806R
M2_1_2cy_28cy	MiSeq	ACTCGATAGT	Mock	PEP	45	No	806R_NI
M2_2_2cy_28cy	MiSeq	CACGAGATGA	Mock	PEP	45	No	806R_NI
M2_3_2cy_28cy	MiSeq	TATAGAGATC	Mock	PEP	45	No	806R_NI
M1_1_55c_28_8	MiSeq	TCTAGCGTGG	Mock	TAS	55	No	806R
M1_2_55c_28_8	MiSeq	TCTCGGATAG	Mock	TAS	55	No	806R
M1_3_55c_28_8	MiSeq	GTATAACGCT	Mock	TAS	55	No	806R
S1.1.2cy.28cy	MiSeq	TCATATCGCG	Sed	PEP	45	No	806R
S1.2.2cy.28cy	MiSeq	TGCGAGACGT	Sed	PEP	45	No	806R
S1.3.2cy.28cy	MiSeq	TACTGCAGCG	Sed	PEP	45	No	806R
s1.1.45cy.28.8cy	MiSeq	GACAGCAAGC	Sed	TAS	45	No	806R
s1.2.45cy.28.8cy	MiSeq	AAGTACACTC	Sed	TAS	45	No	806R
s1.3.45cy.28.8cy	MiSeq	AGTGGCAGGT	Sed	TAS	45	No	806R
S1e.1.2cy.28cy	MiSeq	GCACGCGTAT	Sed	PEP	45	Yes	806R
S1e.2.2cy.28cy	MiSeq	ACTAGCTGTC	Sed	PEP	45	Yes	806R
S1e.3.2cy.28cy	MiSeq	CGAGCTAGCA	Sed	PEP	45	Yes	806R
S2.1.2cy.28cy	MiSeq	TCATCATGCG	Sed	PEP	45	No	806_NI
S2.2.2cy.28cy	MiSeq	ACGTGCTCTG	Sed	PEP	45	No	806_NI
S2.3.2cy.28cy	MiSeq	TACATGATAG	Sed	PEP	45	No	806_NI
S2e.1.2cy.28cy	MiSeq	AGAGTCGCGT	Sed	PEP	45	Yes	806_NI
S2e.2.2cy.28cy	MiSeq	GATATATGTC	Sed	PEP	45	Yes	806_NI
S2e.3.2cy.28cy	MiSeq	ATCATATCTC	Sed	PEP	45	Yes	806_NI
Ma1	PGM	MID-19R	Mock	PEP	30	Yes	806R
Ma2	PGM	MID-20R	Mock	PEP	35	Yes	806R
Ma3	PGM	MID-21R	Mock	PEP	40	Yes	806R
Ma4	PGM	MID-22R	Mock	PEP	45	Yes	806R
Ma5	PGM	MID-23R	Mock	PEP	50	Yes	806R
Ma6	PGM	MID-24R	Mock	PEP	55	Yes	806R
Ma1D	PGM	MID-27R	Mock	PEP	30	Yes	806R
Ma2D	PGM	MID-28R	Mock	PEP	35	Yes	806R
Ma3D	PGM	MID-29R	Mock	PEP	40	Yes	806R
Ma4D	PGM	MID-30R	Mock	PEP	45	Yes	806R
Ma5D	PGM	MID-31R	Mock	PEP	50	Yes	806R
Ma6D	PGM	MID-32R	Mock	PEP	55	Yes	806R
Mb1	PGM	MID-51R	Mock	PEP	30	Yes	806R
Mb2	PGM	MID-52R	Mock	PEP	35	Yes	806R

Mb3	PGM	MID-53R	Mock	PEP	40	Yes	806R
Mb4	PGM	MID-54R	Mock	PEP	45	Yes	806R
Mb5	PGM	MID-55R	Mock	PEP	50	Yes	806R
Mb6	PGM	MID-56R	Mock	PEP	55	Yes	806R
Mb1D	PGM	MID-59R	Mock	PEP	30	Yes	806R
Mb2D	PGM	MID-60R	Mock	PEP	35	Yes	806R
Mb3D	PGM	MID-61R	Mock	PEP	40	Yes	806R
Mb4D	PGM	MID-62R	Mock	PEP	45	Yes	806R
Mb5D	PGM	MID-63R	Mock	PEP	50	Yes	806R
Mb6D	PGM	MID-64R	Mock	PEP	55	Yes	806R
Mc1	PGM	MID-83R	Mock	TAS	30	No	806R
Mc2	PGM	MID-84R	Mock	TAS	35	No	806R
Mc3	PGM	MID-85R	Mock	TAS	40	No	806R
Mc4	PGM	MID-86R	Mock	TAS	45	No	806R
Mc5	PGM	MID-87R	Mock	TAS	50	No	806R
Mc6	PGM	MID-88R	Mock	TAS	55	No	806R
Mc1D	PGM	MID-91R	Mock	TAS	30	No	806R
Mc2D	PGM	MID-92R	Mock	TAS	35	No	806R
Mc3D	PGM	MID-93R	Mock	TAS	40	No	806R
Mc4D	PGM	MID-94R	Mock	TAS	45	No	806R
Mc5D	PGM	MID-95R	Mock	TAS	50	No	806R
Mc6D	PGM	MID-96R	Mock	TAS	55	No	806R
A1	PGM	MID-1R	Chin	PEP	30	Yes	806R
A2	PGM	MID-2R	Chin	PEP	35	Yes	806R
A3	PGM	MID-3R	Chin	PEP	40	Yes	806R
A4	PGM	MID-4R	Chin	PEP	45	Yes	806R
A5	PGM	MID-5R	Chin	PEP	50	Yes	806R
A6	PGM	MID-6R	Chin	PEP	55	Yes	806R
A1D	PGM	MID-10R	Chin	PEP	30	Yes	806R
A2D	PGM	MID-11R	Chin	PEP	35	Yes	806R
A3D	PGM	MID-13R	Chin	PEP	40	Yes	806R
A4D	PGM	MID-14R	Chin	PEP	45	Yes	806R
A5D	PGM	MID-15R	Chin	PEP	50	Yes	806R
A6D	PGM	MID-16R	Chin	PEP	55	Yes	806R
B2	PGM	MID-36R	Chin	PEP	35	Yes	806R
B3	PGM	MID-37R	Chin	PEP	40	Yes	806R
B4	PGM	MID-38R	Chin	PEP	45	Yes	806R
B5	PGM	MID-39R	Chin	PEP	50	Yes	806R
B6	PGM	MID-40R	Chin	PEP	55	Yes	806R
B1D	PGM	MID-43R	Chin	PEP	30	Yes	806R
B2D	PGM	MID-44R	Chin	PEP	35	Yes	806R
B3D	PGM	MID-45R	Chin	PEP	40	Yes	806R
B4D	PGM	MID-46R	Chin	PEP	45	Yes	806R
B5D	PGM	MID-47R	Chin	PEP	50	Yes	806R
B6D	PGM	MID-48R	Chin	PEP	55	Yes	806R
