# Supplementary material for: Deconstructing the Polymerase Chain Reaction: Understanding and Correcting Bias Associated with Primer Degeneracies and Primer-Template Mismatches
Source: PLoS One. 2015 May 21;10(5):e0128122. doi: 10.1371/journal.pone.0128122 (PMC4440812; doi:10.1371/journal.pone.0128122)
Supplement: S4 File — (PDF) [file pone.0128122.s009.pdf]

Supporting Information

S4 File: Temperature gradient tests of PEX and TAS methods using mock community DNA ("Chin") at varying annealing temperatures.

Method	Annealing Temperature	Exonuclease	Reverse Primer	Average percent abundance of reads mapping to references				Ideal Score	Ideal Score	Ideal Score
Stage "A" Annealing Temperature, PEX PCR Method				Mock A	Mock B	Mock C	Mock D	4 targets	3 targets	2 targets
PEX PCR	30	Yes	806R	30.40	58.19	11.12	0.29	77*	50*	31 NS
PEX PCR	35	Yes	806R	15.49	57.76	26.36	0.39	68*	49*	58
PEX PCR	40	Yes	806R	15.46	51.70	32.29	0.56	68*	38*	54 NS
PEX PCR	45	Yes	806R	32.85	35.00	31.56	0.59	49*	4*	3*
PEX PCR	50	Yes	806R	32.62	35.23	31.93	0.23	50*	4*	4*
PEX PCR	55	Yes	806R	29.68	41.73	28.44	0.15	50*	17*	17*
Stage "A" Annealing Temperature, TAS PCR Method										
TAS PCR	30	No	806R	33.26	63.77	2.94	0.04	94	61	31
TAS PCR	35	No	806R	27.89	65.93	6.11	0.08	88	65	41 #
TAS PCR	40	No	806R	21.06	70.54	8.34	0.06	91	74	54
TAS PCR	45	No	806R	34.58	58.95	6.42	0.05	87	54	26
TAS PCR	50	No	806R	29.81	63.35	6.78	0.06	86	60	36
TAS PCR	55	No	806R	18.66	76.33	4.93	0.08	103	86	61

* Significant decrease in PEX PCR method relative to TAS PCR method; p<0.03, two-tailed TTEST (unequal variance)

Significant decrease in TAS PCR relative to PEX PCR method; p<0.03, two-tailed TTEST (unequal variance)

NS = Not significant, two-tailed TTEST (unequal variance)
